# Sumoylation-deficient Prdx6 repairs aberrant Sumoylation-mediated Sp1 dysregulation-dependent Prdx6 repression and cell injury in aging and oxidative stress

**DOI:** 10.18632/aging.101547

**Published:** 2018-09-12

**Authors:** Bhavana Chhunchha, Eri Kubo, Prerna Singh, Dhirendra P. Singh

**Affiliations:** 1 Department of Ophthalmology and Visual Sciences, University of Nebraska Medical Center, Omaha, NE 68198, USA; 2 Department of Ophthalmology, Kanazawa Medical University, Ishikawa 920-0293, Japan

**Keywords:** oxidative stress, Sumo1, Senp1, Sp1, Prdx6, antioxidant, cell survival

## Abstract

Progressive deterioration of antioxidant response in aging is a major culprit in the initiation of age-related pathobiology induced by oxidative stress. We previously reported that oxidative stress leads to a marked reduction in transcription factor Sp1 and its mediated Prdx6 expression in lens epithelial cells (LECs) leading to cell death. Herein, we examined how Sp1 activity goes awry during oxidative stress/aging, and whether it is remediable. We found that Sp1 is hyper-Sumoylated at lysine (K) 16 residue in aging LECs. DNA binding and promoter assays revealed, in aging and oxidative stress, a significant reduction in Sp1 overall binding, and specifically to Prdx6 promoter. Expression/overexpression assay revealed that the observed reduction in Sp1-DNA binding activity was connected to its hyper-Sumoylation due to increased reactive oxygen species (ROS) and Sumo1 levels, and reduced levels of Senp1, Prdx6 and Sp1. Mutagenesis of Sp1 at K16R (arginine) residue restored steady-state, and improved Sp1-DNA binding activity and transactivation potential. Extrinsic expression of Sp1K16R increased cell survival and reduced ROS levels by upregulating Prdx6 expression in LECs under aging/oxidative stress, demonstrating that Sp1K16R escapes the aberrant Sumoylation processes. Intriguingly, the deleterious processes are reversible by the delivery of Sumoylation-deficient Prdx6, an antioxidant, which would be a candidate molecule to restrict aging pathobiology.

## Introduction

All cells or organisms encounter many types of environmental stresses and apoptotic stimuli. In young cells, the defense responses are efficient, but with age the responses are weakened by dysregulation of antioxidant response, which leads to impaired cellular function and increased vulnerability [1,2]. Protein homeostasis is essential to maintain cellular systems during the progressive rise of oxidative stress in aging [3–5]. Many biologically relevant factors in the cellular and external environments, such as chemical, ultraviolet B (UVB) radiation, H_2_O_2_ and growth factors, have been shown to initiate reactive oxygen species (ROS)-evoked adverse signaling in cells due to malfunction of antioxidant defense system. The antioxidant defense system is equipped with antioxidant proteins, like CAT, SODs, GPX1, Prdxs. The stress factors significantly negatively alter their activity that results in age-related pathobiology [6]. We and other investigators have reported that Prdx6 maintains cellular homeostasis by maintaining calcium homeostasis and cell membrane and DNA integrity by optimizing ROS levels against the stressors such as H_2_O_2_, UVB, paraquat, endoplasmic reticulum (ER) stress and overstimulated growth factors [7–9]. Our published report has also shown that expression of the antioxidant defense gene Prdx6 and its regulator Sp1 decline significantly in an age-dependent manner, which is directly associated with increased production of ROS and cell death in human lens epithelial cells (hLECs) [10,11].

The protective protein Prdx6 is a member of the Prdx family, which consists of conserved single cysteine (Cys) residue (known as 1-Cysteine). This has both GSH-Peroxidase and acidic Ca^2+^-independent phospholipase A_2_ (PLA_2_) activities, and is widely expressed at significant levels in eye lens, lung, liver, testis and brain. Prdx6 is predominantly localized in cytoplasm, including endoplasmic reticulum, mitochondria, lysosomes and plasma membrane [5,7,12–15]. This localization pattern of Prdx6 in ROS-producing organelles emphasizes its biologically important function in maintaining redox homeostasis. We have shown that Prdx6 expression is significantly reduced in aging lenses and LECs [8,16], and these LECs/lenses are highly susceptible to cell death and lens opacity induced by stressors [5,17–19]. In the current study, we found that TATA-less human Prdx6 promoters bore four active binding sites for Sp1, which are evolutionarily well conserved (This study, Table 1). Transcription factor Sp1 belongs to the family of Sp/KLF (Kruppel-Like Factor) nuclear proteins [20,21]. Sp1 is responsible for basal transcription of genes, and can also modulate its target gene transcription in response to physiological and pathological stimuli [22]. It binds with high affinity to GC-rich motifs (such as 5’-GGGGCGGGG-3’ or 5’-G/T-GGGCGG-G/A-C/T-3’ or 5’-G/T-G/A-GGCG-G/T-G/A-G/A-C/T-3’) and regulates the expression of TATA- containing or TATA-less genes via protein-protein, protein-DNA interaction or interplay with other transcription factors, and also through a large number of genes involved in a variety of cellular physiological processes such as growth, apoptosis, differentiation and senescence [23]. Nonetheless, posttranslational modifications of proteins are an important mechanism that regulates protein function, activity and localization [24]. Posttranslational modifications of Sp1, such as glycosylation, phosphorylation, methylation, acetylation, ubiquitination, and Sumoylation modulate the Sp1 and its target gene expression by affecting its protein level, transcription and DNA-binding activities [20,25–27]. Oxidative stress-induced aberrant protein modification has been implicated in the etiology and progression of many human diseases [2,28–31]. Sp1 expression has been shown to be significantly reduced in aging cells [11] and cells facing oxidative stress [5,9,11,32]. Cells overexpressing Sumo display repressed expression of Sp1 and its target gene Prdx6, whereas Sumo-protease (Senp1) overexpression enhances Prdx6 expression and transcription activity by increasing the levels of Sp1 [5]. In this scenario, we postulated that aberrant Sumoylation of Sp1 during aging and oxidative stress appears to be a plausible cause for Sp1 dysregulation and modulation of its target genes.

**Table 1:** Conserved Sp1 response elements in TATA-less Prdx6 promoter of mouse, rat and human.

**Species**	**Sp1 binding sequences**	**Position**	**Strand(s)**	**References**
**GC-Box-1**				
Mouse	ngCCCGCCCGn	-19/27	(+)	Chhunchha et al., 2011
Rat	ngCCCGCCCGn	-33/41	(+)	-
Human	nCCCCGCCCCn	-53/60	(+)	This study
**GC-Box-2**				
Mouse	ngCCCCGCCCan/	-61/69	(+)	Chhunchha et al., 2011
Rat	ngCCCCGCCCcn	-71/80	(+)	-
	ngCCCCGCCCcn			
Human	ngCCCCGCCCcn	-86/93	(+)	This study
**GC-Box-3**				
Mouse	naCCCCGCCCcn	-82/89	(+)	Chhunchha et al., 2011
Rat	ngCCCCGCCCcn	-112/119	(+)	-
Human	ngCCCCGCCCcn/	-134/141	(+)	This study
	ngCCCCGCCCcn	-139/146	(+)	This study
**GC-Box-4**				
Human	ngCCCCGCCCcn	-156/163	(+)	This study

Multiple sequence alignment (by CLUSTALW) of mouse, rats and human Prdx6 gene promoter showing that Sp1 response element (GC-box) is well-conserved. Sp1 response elements in Prdx6 promoter are predicted by MatInspector (Genomatix-software suit), TFsearch and TEESS websites, and aligned using CLUSTALW, Clustal Omaga (www.ebi.ac.uk/TOOLs/services) and PRRN (www.genome.jp/tmp/prrn).

Sumos play important roles in the regulation of diverse cellular processes, including gene transcription, protein stabilization, and subcellular localization [12,33–35]. Most, if not all, Sumo modification of transcription factors results in repression of their activity. Three major Sumo isoforms, Sumo1, Sumo2 and Sumo3 have been identified in mammals [24,26]. Sumo1 exhibits ~50% amino acid sequence identity with Sumo2 and Sumo3, whereas Sumo2 and Sumo3 share about 97% amino acid sequence identity and are referred to as Sumo2/3 in most cases [24,33,36–40]. Sumo binds covalently to €-lysine embedded in the ɸKXE motif (ɸ represents hydrophobic amino acid, and X stands for any amino acids) within the target protein in an enzymatic cascade reaction leads to protein Sumoylation [24,40]. Sumoylation can be reversed by a family of sentrin/Sumo-specific proteases (Senps) [41,42]. Senp1 is largely responsible for the deconjugation of both Sumo1 and Sumo2/3 modifications, and regulates the activity of many transcription factors including Sp1 *in vivo* and *in vitro* [5,11,12,41,43]. This process can be aberrantly affected during oxidative stress and aging, leading to aberrant Sumoylation processes of proteins like Sp1, and thereby altering protein functions (dysregulation of Sp1 activity in the current study).

In the study reported here, we observed that during aging and oxidative stress, a progressive decline of Prdx6 expression was linked to an increase of Sp1 Sumoylation with decrease in Sp1 expression wherein Sp1-DNA binding activity to Prdx6 promoter was greatly reduced. We also noted that reduction in Sp1-DNA binding activity was connected to increased Sumo1 and ROS levels, and decreased Senp1 and Prdx6 as well as reduction in Sp1-DNA activity and expression in aging LECs and cells facing oxidative stress. We found that Sp1 was Sumoylated *in vivo* at K16 residue in LECs, a major site for the Sumoylation of Sp1. Additionally, data revealed that overexpression of Sumoylation–deficient Sp1K16 improved DNA-binding activity by escaping the erratic Sumoylation that occurs in aging or oxidative stress. An important observation was that delivery to cells of Prdx6 mutant at Sumo1 motif(s) linked to TAT-transduction domain provided cytoprotection by restoring Sp1 stability and DNA-binding activity and protecting against oxidative cell injury by halting ROS-driven aberrant Sumoylation processes. The findings offer a new perspective for developing antioxidant Prdx6-based therapy to rescue cells and organisms from ROS-evoked aberrant Sumoylation signaling.

## RESULTS

### Age-related increases of ROS levels in LECs were connected to progressive decline in Sp1 and Prdx6 expression and Sp1-DNA binding activity to its GC rich elements

**Figure 1:**
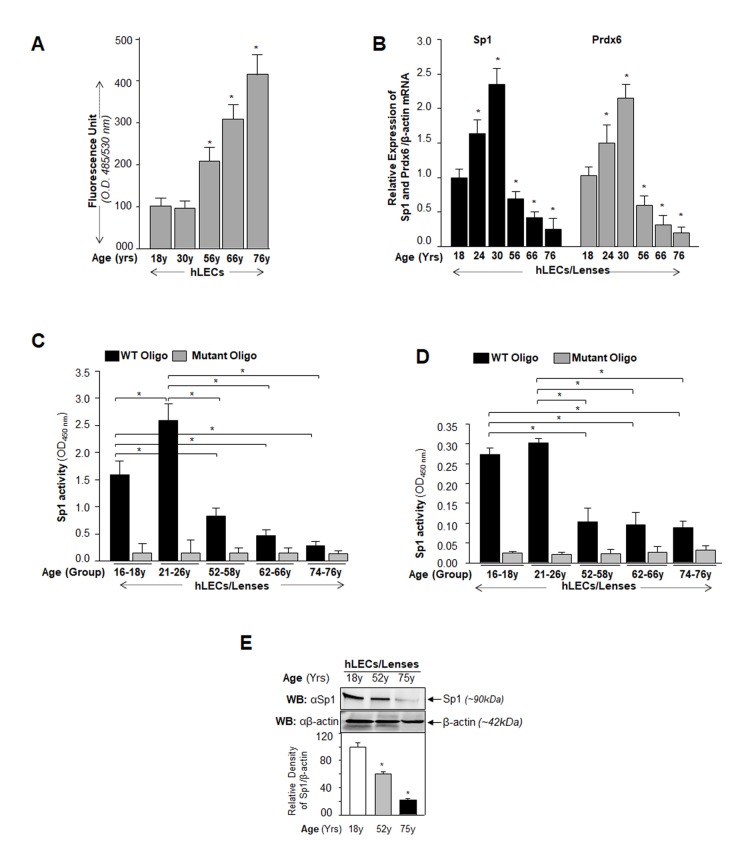
**Aging human LECs/lenses showing elevated levels of ROS and progressive decline in Sp1 and Prdx6 expression connected to reduction in Sp1 activity.** (**A**) ROS levels increased progressively in aging hLECs. Cells were cultured in 96 well plate (5000/well), and ROS were quantified using H2-DCF-DA dye assay as shown. Data represent the mean ± S.D. of two independent experiments. Younger (18y) vs aging samples; *p < 0.001. (**B**) Aging hLECs displayed progressive decline in levels of Sp1 and its target gene, Prdx6 mRNA. Total RNA was isolated from human LECs/ lenses of different ages as indicated and was processed for real-time PCR analysis with specific primers. The data represent the mean ± S.D. from three independent experiments. *p* values were determined for younger vs aging samples. *p < 0.001. (**C**) Aging/aged human lenses/LECs displayed significant loss of Sp1 activity. Nuclear extracts prepared from aging/aged hLECs/lenses were used for assay. LECs/lenses were divided into five age groups: 16-18y (n=6); 21-26y (n=6); 52-58y (n=8); 62-66y (n=8); 74-76y (n=8). Nuclear extracts containing equal amounts of protein were processed and assayed for Sp1 activity using a commercially available kit (Active motif) as described in Materials and Methods. The data represent the mean ± S.D. from three independent experiments. *p* values were determined for younger vs aging samples. *p < 0.001. (**D**) Nuclear extracts containing equal amounts of proteins were processed for Sandwich ELISA to measure the total Sp1 protein. Total Sp1 proteins were equalized with the O.D. of Sandwich ELISA and processed for Sp1 transactivation assay using a commercially available kit (Active motif) as ascribed in Materials and Methods. P value were determined for younger vs aging samples. *p < 0.001. (**E**) Aging hLECs showing significant loss of Sp1 protein. Cellular proteins were isolated from hLECs and human lenses of different ages as described in Materials and Methods section, and as indicated. An equal amount of protein was loaded onto SDS-PAGE, and immunoblotted using Sp1 antibody. Upper panel; expression levels of Sp1, Lower panel; membrane probed with β-actin antibody as loading/internal control. Each band of blot was quantified using densitometer shown below. Images are representatives from three independent experiments. *P* value were determined for younger vs aging samples. *p < 0.001.

During aging, gene expression levels change, a situation which may be associated with the accumulation of high levels of ROS [44]. To determine a connection between levels of ROS, Prdx6 and Sp1, and binding efficiency of Sp1 to its response elements (GC-box), we first monitored the intracellular redox-state of primary hLECs of different ages. Quantification by staining with H_2_DCFDA dye showed an age-dependent progressive increase in ROS levels (Fig. 1A), which reached significantly higher levels in aged hLECs (Fig. 1A, 56y onward). Next, we isolated RNA from the same groups of aging cells and quantified mRNA by real-time PCR. We observed that the levels of both Sp1 and Prdx6 mRNA in hLECs declined with aging, and this loss was more significant in aged cells (Fig. 1B, 56y onward). Collectively the results revealed a significant inverse correlation between expression of Sp1/Prdx6 and ROS levels during aging. Because we found a direct correlation between expression levels of Prdx6 mRNA and its regulator Sp1 mRNA and protein (Fig. 1), we surmised that this could be related to a loss of Sp1 cellular abundance or reduction in its binding efficiency to Prdx6 promoter due to increased levels of ROS in aging cells. To explore that possibility, nuclear protein isolated from hLECs of different ages was used to quantify the presence of active Sp1 by using TransAM Sp1 transcription factor assay (Active Motif) as well as Sp1 protein level. Data revealed that, indeed, Sp1-DNA activity declined (Fig.1C), and that reduction in Sp1-DNA activity was connected to decline of Sp1 cellular levels with increase in age (Fig. 1E), suggesting that an increase in ROS-induced oxidative stress could jeopardize Sp1 activity and lead to repression of Prdx6 mRNA. Figure 1E reveals that Sp1 protein declined with advancing age as evidenced by Western analysis. However, due to the limited supply of primary hLECs, we were able to perform Sp1 protein expression analysis on only cells of certain age points (as Western analysis requires larger amounts of protein extracts). Next we asked whether dysregulation of Sp1 was due solely to reduced cellular abundance or if a reduction in Sp1 binding efficiency in nuclear extracts of aging cells might have made a contribution. We equalized Sp1 contents in nuclear extracts of hLECs isolated from different age groups using Sp1 specific sandwich-ELISA as described in Materials and Methods, and measured the Sp1/ DNA binding activity with TransAM Sp1 transcription factor assay (Active Motif). We found that both reduced abundance and a decrease of Sp1 binding efficiency were responsible for dysregulation of Sp1-DNA binding activity during aging (Fig. 1D).

### *In vivo* DNA binding assay revealed reduction in Sp1-DNA binding to Prdx6 promoter during H_2_O_2_ or UVB-induced oxidative stress in LECs

**Figure 2:**
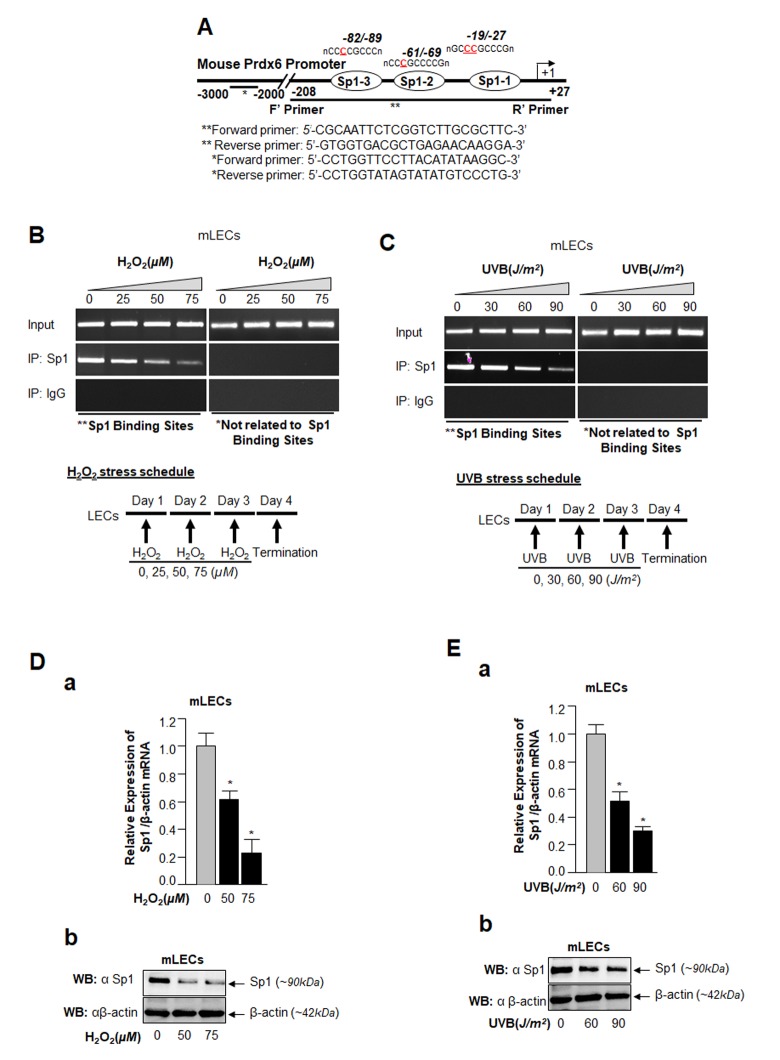
**ChIP analysis of genomic DNA from LECs facing oxidative stress disclosed a significant loss in Sp1-DNA binding to Prdx6 gene promoter.** (**A**) Schematic illustration of 5’-proximal promoter region of Prdx6 containing Sp1 binding sites showing primer location and sequences used in ChIP assay. (**B** and **C**) ChIP assay showing Sp1 binding to Prdx6 promoter *in vivo*. Chromatin samples were prepared from *Prdx6^+/+^* LECs (mLECs) exposed to different doses of H_2_O_2_ (0, 25, 50 and 75µM) and/or UVB (0, 30, 60 and 90J/m^2^) as indicated. 72h later samples were subjected to ChIP assay with ChIP grade antibodies, anti-Sp1 or IgG control. The DNA fragments were amplified by using primers designed to amplify −208 to +27 region of the Prdx6 promoter bearing Sp1 sites (**) and contiguous sequence (−2229 to −2356) to which Sp1 does not bind (*) as indicated. PCR products were resolved onto agarose gel and visualized with ethidium bromide staining. Photographs are representative of three experiments. (**D** and **E**) Expression assays showing H_2_O_2_- and UVB- induced declined expression of Sp1 in mLECs. mLECs cells were treated with different concentrations of H_2_O_2_ (**D**) and/or UVB (**E**) multiple time for 3 days as indicated. Total RNA and protein were isolated and subjected to real-time PCR and Western analysis with Sp1 specific probes, respectively. Data revealed a concentration –dependent reduced expression of Sp1 mRNA (**Da** and **Ea**; Gray vs black bars; *p<0.001) and protein (**Db** and **Eb**).

**Figure 3:**
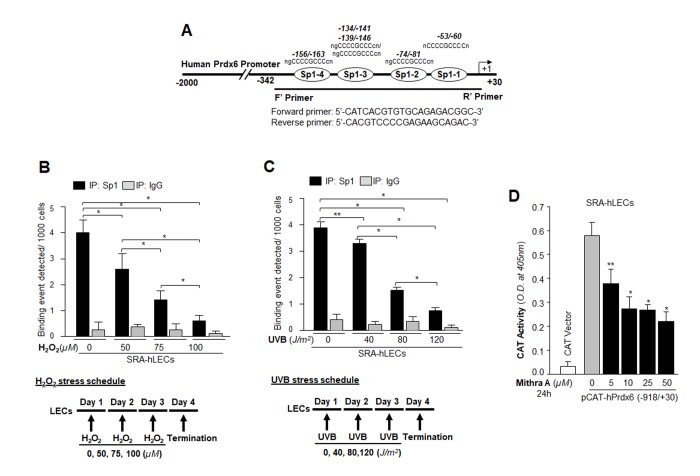
**Oxidative stress attenuated Sp1 binding to its GC-Box elements present in hPrdx6 gene promoter.** **Table 1****.** Evolutionary conserved Sp1 binding sequences in TATA-less Prdx6 promoters of mouse, rat and human cells. (**A**) Schematic illustration of 5’-proximal promoter region of Prdx6 containing Sp1 (GC-Box) binding sites showing primer location and sequences used in ChIP assay. (**B** and **C**) Oxidative stress (H_2_O_2_ or UVB)-induced reduction in DNA binding activity of Sp1 to hPrdx6 gene promoter containing GC-Box (Sp1 sites) in SRA-hLECs. ChIP assay was carried out by using ChIP-IT® Express and ChIP-IT® qPCR analysis kits (Active motif). Chromatin samples prepared from SRA-hLECs were exposed to varying concentrations of H_2_O_2_ (0, 50, 75 and 100µM) or UVB (0, 40, 80 and 120J/m^2^), and were subjected to ChIP assay with ChIP grade antibodies, anti-Sp1 (black bars) and control IgG (gray bars). The DNA fragments were used as templates for qPCR by using primer designed to amplify -342 to +30 region of the human *Prdx6* gene promoter bearing GC-box (Sp1 sites). Histogram showed the amplified DNA by qPCR analysis: (**B**) Control (0) vs 50µM vs 75µM vs 100µM H_2_O_2_ treatment. (**C**) Control (0) vs 40J/m^2^ vs 80J/m^2^ vs 120J/m^2^ UVB exposure. The data represent mean ± SD from three independent experiments (**p<0.05; *p<0.001). (**D**) Human Prdx6 promoter activity inhibited by mithramycin A (Mithra A), an inhibitor of Sp1, validated Sp1 regulation of hPrdx6 gene. Cells were transfected with CAT-hPrdx6 (-918/+30) or empty CAT vector construct and treated with Mithra-A at different concentrations for 24h. Data represent mean ± SD from three independent experiments (**p<0.05; *p<0.001).

Major causes of age-related pathobiology include an increased burden of oxidative stress and its associated damage to cells/tissues due to accumulation of ROS, a progressive decline of antioxidant expression, and a deterioration of transcription factor activity in aging [10,45–47]. Our previous report [5] and an analysis of data presented in Figure 1 demonstrate that a progressive increase of ROS levels in aging cells was linked to progressive reduction in expression and activity of Sp1 and its target gene Prdx6 mRNA. We surmised that there might be a decrease in Sp1 interaction with its elements present in Prdx6 promoter due to increase of oxidative load *in vivo*. To examine this possibility, we performed ChIP assay using antibody specific to Sp1, as described in Materials and Methods, using hLECs facing different concentrations of oxidative stress. Reports by others using other cell types have shown that H_2_O_2_-induced oxidative stress lowers Sp1-DNA binding [48,49]. We initially performed bioinformatics analysis and spotted out Sp1 binding sites in mouse, rat and human gene promoters. As shown in Table 1, Sp1 binding sites present in mouse, rat and human Prdx6 promoter are well conserved. Next, chromatin samples were prepared from mouse and human LECs exposed to H_2_O_2_ or UVB multiple times as indicated in Figs. 2 and 3. Protein-DNA complex was pulled down with anti-Sp1 antibody or with control IgG. Immunoprecipitated complex was processed for RT-PCR (mLECs) and qPCR (hLECs) using the primer that encompassed mouse and human Prdx6 promoters containing Sp1-responsive elements (Figs. 2A and 3A). We observed a significant decrease in the enrichment of Sp1 with its target elements at Prdx6 promoters in samples exposed to both H_2_O_2_ (Figs. 2B and 3B) and UVB (Figs. 2C and 3C). Interestingly, that decrease of Sp1 binding was directly related to decline of Sp1 mRNA and protein as revealed by expression analysis, suggesting that oxidative stress-induced reduced cellular abundance of Sp1 was one cause of decrease in Sp1-DNA activity (Fig. 2D). No effect was observed on internal control as shown in Fig. 2D and E, as observed in aging hLECs (Fig. 1E). Furthermore, in the same experiment, qPCR analysis of Sp1 targeted gene, Prdx6 transcription also showed a decline in Prdx6 mRNA, which was directly related to a decrease of Sp1-DNA binding (as observed in the ChIP experiment), corroborating that aging or oxidative stress did not affect gene regulation globally and transcriptional machinery was in active state, at least in hLECs facing oxidative stress in the model system observed. Sp1 is a transregulator of mouse Prdx6 transcription [5,9]. To examine whether Sp1 binding to human Prdx6 promoter activated the promoter through its responsive elements (GC-boxes), we performed transactivation assay by using mithramycin A (Mithra A), an inhibitor of Sp1 (Fig. 3D). SRA-hLECs were transiently transfected with pCAT-hPrdx6 (-918/+30) promoter or empty CAT vector, and treated with increasing amounts of Mithra-A as indicated in Fig. 3D. We observed that Mithra-A inhibited Prdx6 promoter activity in dose-dependent fashion, suggesting that Sp1 bound and activated human Prdx6 promoter. Taken together, our results revealed that oxidative stress attenuated Prdx6 transcription through Sp1.

### *Prdx6-deficient* LECs, a model for aging, displayed aberrant Sumoylation of Sp1 during oxidative stress

**Figure 4:**
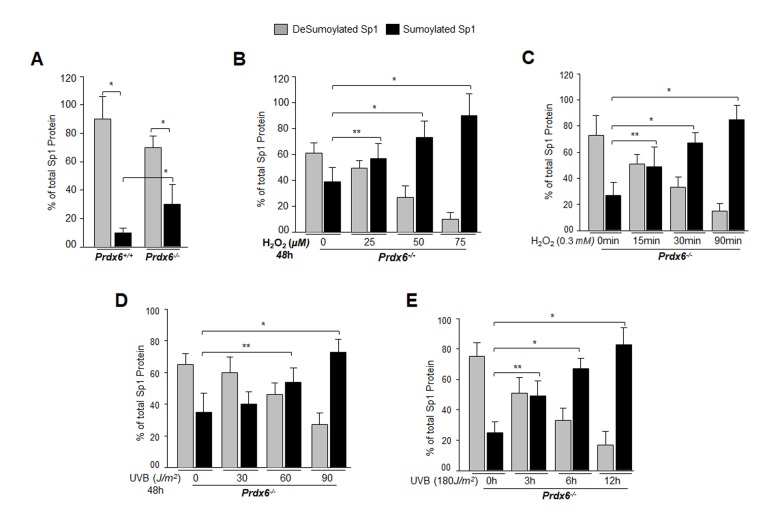
***Prdx6^-/-^* LECs, a model for aging, bore an enhanced Sumoylated form of Sp1, and levels were further increased with exposure to oxidative stress.** (**A**) Nuclear extracts were prepared from *Prdx6^+/+^* and *Prdx6^−/−^* mLECs and submitted to Sp1 Sandwich/Sumo1-ELISA assays to examine the total and Sumoylated forms of Sp1 protein. Sumoylated Sp1 protein was subtracted from total Sp1 protein, and results are presented as deSumoylated (gray bars) and Sumoylated (black bars) forms of Sp1. The data represent mean ± SD from three independent experiments. *Prdx6^+/+^* vs *Prdx6^−/−^* ; *p<0.001. (**B**-**E**) *Prdx6^-/-^* LECs displayed increased levels of Sumoylated Sp1 in response to increased oxidative loads. *Prdx6^-/-^* LECs were exposed to H_2_O_2_ (**B** and **C**) or UVB (**D** and **E**) in different concentrations and for different time intervals as indicated. Nuclear extracts were prepared and used to perform Sp1 sandwich/Sumo1-ELISA specific to Sp1. Sumoylated Sp1 protein was subtracted from total Sp1 protein, presented as deSumoylated Sp1 (gray bars) and Sumoylated Sp1 (black bars). B-E: Data represent mean ± SD from three independent experiments. 0µM vs 25, 50 and 75µM (H_2_O_2_), 0 min vs 15 , 30 and 90 min of H_2_O_2_ exposure and 0 J/m^2^ vs 30, 60 and 90 J/m^2^ (UVB) and 0h vs 3, 6 and 12h UVB exposure; **p<0.05; *p<0.001.

From the previous experiments, it was clear that DNA binding activity of Sp1 declined, but it was not clear how and by what mechanism the dysregulation occurred. Our previous findings had shown a significantly global aberrant increase in Sumo1 conjugation of proteins in *Prdx6^-/-^* LECs (a model for aging) as well as in aging hLECs, and that increase was linked to a progressive increase of ROS-induced oxidative stress [5]. These LECs also showed reduced Sp1, Prdx6 and Senp1 expression (as well as the presence of an inactive dimeric form of Senp1) with increased Sumo1 levels, suggesting oxidative-induced aberrant Sumoylation signaling as a possible cause of reduction in Prdx6 and Sp1 levels. To uncover the role of Sumo1 in dysregulation of Sp1 during oxidative stress, we analyzed deSumoylation and Sumoylation status of Sp1 in *Prdx6^+/+^* and *Prdx6^-/-^* LECs with or without oxidative stress by using Sp1 sandwich/Sumo1-ELISA as shown in Fig. 4A. We observed a significantly higher level of the Sumoylated form of Sp1 in *Prdx6^-/-^* compared to *Prdx6^+/+^* LECs, suggesting that *Prdx6* deficiency (increased ROS levels) may cause an increase in Sumoylation status of Sp1, at least in LECs. In an earlier report [5], we showed that *Prdx6^-/-^* LECs facing oxidative stress displayed increased Sumo1 conjugation and reduced Sp1 expression. Furthermore, *Prdx6^-/-^* LECs exposed to different concentrations of H_2_O_2_ for 48h (Fig. 4B) or 0.3mM H_2_O_2_ for different time intervals (Fig. 4C) displayed dramatic increases in Sp1 Sumoylation in dose- and time-dependent manner. Similarly, we exposed *Prdx6^-/-^* LECs to UVB (to see indirect effect) as eyes are maximally exposed to UVB as indicated (Fig. 4D and 4E). At 60 and 90J/m^2^ UVB exposure, Sp1 Sumoylation significantly increased, but at 30J/m^2^ no significant change was detected (Fig. 4D). At the higher dose of 180J/m^2^ UVB, we observed a further significant increase in Sp1 Sumoylation in time-dependent fashion (Fig. 4E). Data revealed that as *Prdx6*-deficient mLECs came under redox stress, they showed increased Sumo1 and increased Sp1 Sumoylation. Oxidative stress induced by UVB or H_2_O_2_ increased production of ROS in *Prdx6^-/-^* LECs, which may have further increased Sumo1 conjugation and thereby reduced Sp1 expression by increasing the Sumoylation status of Sp1.

### Increases in Sumo1 levels were directly connected to increased Sumoylation of Sp1, and inversely related to Senp1 levels in aging/aged lenses/LECs

**Figure 5:**
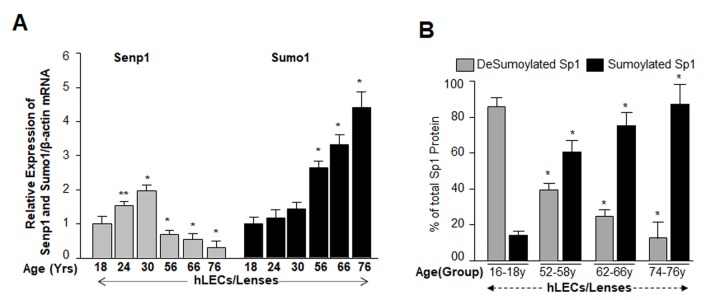
**Aging human LECs/lenses showed age-dependent decline in Senp1 expression and increased Sumo1 expression, which were directly related to increase Sp1 Sumoylation.** (**A**) Aging hLECs/lenses displayed a progressive decline in the deSumoylating agent Senp1 and an increase in Sumo1 levels. Total RNA was isolated from human lenses/LECs of different ages as indicated, and was processed for real-time qPCR analysis as stated in Materials and Methods. The data represent the mean ± S.D. values from three independent experiments. *P* values were determined for younger (18y) vs aging samples. **p<0.05, *p < 0.001. (**B**) Nuclear lysates were prepared from hLECs/lenses of various ages and were submitted to Sp1 sandwich/Sumo1-ELISA to examine the total and Sumoylated Sp1 protein. Primary hLECs isolated from lenses of different ages were divided into four groups: 16-18y (n=6); 52-58y (n=8); 62-66 (n=8); and 74-76y (n=8). Sumoylated Sp1 protein was subtracted from total Sp1 protein, and presented as deSumoylated (B, gray bars) and Sumoylated (B, black bars) forms of Sp1. Data represent mean ± SD from two independent experiments. *p* values were determined for younger (16-18y) vs aging samples. *p < 0.001.

We next examined whether Sumoylation status of Sp1 has any link to level of Sumo1 or Senp1 during aging of hLECs, similar to the relationship observed in Prdx6-deficient mLECs which showed increased levels of ROS. Because of the increased ROS levels (Fig.1A), we posited that aging/aged hLECs should have increased amounts of Sumoylated Sp1. (Fig. 4). To examine this, total RNA isolated from human lenses/LECs of different ages was processed for mRNA analysis using qPCR. Data showed a significant increase in Sumo1 and reduction in Senp1 mRNA with increases in age (Fig. 5A, 56y onward). To determine whether modulation of Sumo1 or Senp1 expression in aging LECs had any influence on Sumoylation or deSumoylation status of endogenous Sp1, we quantified levels of Sumoylated and deSumoylated forms of Sp1 in the same group of aging/aged hLECs by utilizing a sensitive Sp1 sandwich/Sumo1-ELISA as described in Materials and Methods. Equal amounts of nuclear extracts isolated from hLECs were processed. Data revealed that the ratio of deSumoylated and Sumoylated forms of Sp1 was 4:1 in the youngest age group (16-18y). The ratio gradually changed with aging: Age group 52-58y, 1:1.5; Age group 62-66y, 1:3; and Age group 74-76y, 1.4. Taken together, data disclosed that Sumoylation of Sp1 was increased with aging/aged hLECs and suggested that oxidative load might be a prime cause for aberrant Sumoylation of Sp1.

### Sumoylation negatively affected the DNA binding activity of Sp1 while the deSumoylating agent Senp1 enhanced Sp1-DNA binding

**Figure 6:**
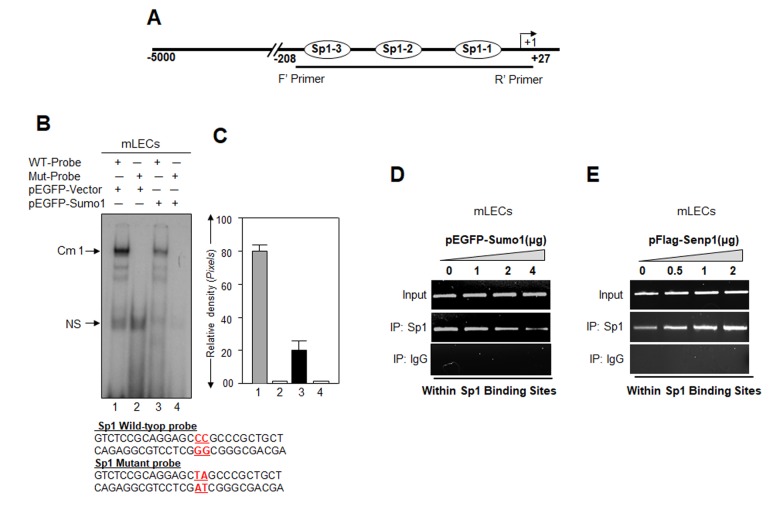
**Cells overexpressing Sumo1 showed reduced Sp1 binding to its responsive elements in Prdx6 promoter.** (**A**) Schematic illustration of Prdx6 gene promoter. (**B**) Gel-shift mobility assay showing that Sumo1 reduced the Sp1 DNA–binding activity of Prdx6 gene promoter. Gel-shift mobility assay was carried out using nuclear extracts isolated from mLECs transfected with pEGFP-Vector (Lanes 1 and 2) or pEGFP-Sumo1 (Lanes 3 and 4) incubated with 32p-labeled wild type probe (Lanes 1 and 3) or its mutant (Lanes 2 and 4). A diminished Cm1 band was observed in cells overexpressing Sumo1 (Lane 3) in comparison to vector control (Lane 1). No binding occurred in mutant probes (Lanes 2 and 4). (**C**) Histogram represents densitometry analysis of DNA-protein complex formed in gel-shift assay. Lane 1 vs lane 3, *p < 0.001. (**D**) ChIP assay showing Sumo1 overexpression significantly suppressed Sp1-DNA binding in Prdx6 gene promoter in dose-dependent manner. mLECs were transiently transfected with different concentrations of pEGFP-Sumo1 (0, 1, 2 and 4 μg). 72h later ChIP assay was carried out with anti-Sp1 and control IgG antibodies. Pulled DNA fragments were subjected to PCR analysis for Sp1 binding *cis*-elements of *Prdx6* promoter. The product was analyzed through agarose-gel. Data represent three experiments. (**E**) Senp1 overexpression dramatically enhanced Sp1-DNA binding in concentration-dependent -manner. mLECs were transfected with increasing concentrations of pFlag-Senp1 (0, 0.5, 1 and 2μg) for 72h. ChIP analysis was carried out using chromatin samples prepared from transfected LECs with a ChIP grade antibody, anti-Sp1 and control IgG. The DNA fragments were used as templates for PCR by using primers designed to amplify −208 to +27 region of the Prdx6 promoter bearing Sp1 binding sites as shown. PCR product was analyzed through agarose gel as shown.

Studies showed that Sumo1 conjugation might alter the DNA binding of proteins [11,27,50–52]. Our current study (Figs.1 to 5) and previous report [5] revealed that Sumo1 overexpression reduced Sp1 mRNA and protein expression, in contrast to Senp1. Next, to determine whether Sumoylation directly affects Sp1-DNA binding activity in Prdx6 promoter, we carried out a gel-shift mobility assay. Nuclear extracts with equal amount of protein from LECs transfected with pEGFP-Sumo1 or pEGFP-Vector were incubated with radiolabeled WT or mutant probes and processed for gel-shift assay (Fig. 6B). The binding of Sp1 in nuclear extract from Sumo-transfected cells to the WT probe was reduced significantly (Fig. 6B, lane 3; 6C, right panel, densitometry of bands) compared to vector-transfected cells (Fig. 6B, lane 1). To determine if Sumoylation/deSumoylation affects the DNA-binding of Sp1 *in vivo*, we carried out a ChIP assay. mLECs were extrinsically transfected with different concentrations of pEGFP-Sumo1 or pFlag-Senp1. Chromatin samples were prepared as described in Materials and Methods. The Sp1 antibody or control rabbit IgG immunoprecipitated complex was processed and analyzed by qPCR using primers specific to promoter region as shown in Fig. 2A. Fig. 6D shows that Sumo1 overexpression significantly reduced the Sp1-DNA binding in dose-dependent manner with the endogenous *Prdx6* gene, and this binding was increased in cells overexpressing Senp1 (Fig. 6E) in concentration-dependent fashion. There was no detectable Sp1-DNA binding with IgG control immunoprecipitated samples. Sumo1 overexpression significantly reduced the level of Sp1 (Fig. 6B and 6D). In contrast, increased abundance of Sp1 protein was detected in cells overexpressed with Senp1 (Fig. 6E). Taken together, data indicate that increased abundance of Sumo1 dysregulated Sp1 activity.

### K16 residue of Sp1 was a target for Sumo1 conjugation in LECs, and an increased level of Sumo1 affected Sumoylation status of Sp1

Genes/proteins and their functions can differ in different cell types and cell backgrounds. Our initial aim was to confirm the findings of others that Sp1 is a substrate for Sumo1, and it is Sumoylated at K16 residue [23,32,34]. To determine whether Sp1 is indeed Sumoylated at K16 in hLECs in vivo as reported by others, we performed an immunoprecipitation assay using antibody specific to Sp1 with nuclear extract of hLECs overexpressing EGFP-Sumo1 plasmid. We observed that protein precipitated with anti-Sp1 monoclonal antibody, when immunoblotted with anti-Sp1 or anti-Sumo1 polyclonal antibodies as shown in Fig. S1A, revealed the presence of two bands with Sp1 polyclonal antibody (Fig. S1Aa, lanes 1 and 2, endogenous Sp1, ~90kDa; Fig. S1Aa, lane 2, endogenous Sp1 Sumoylated with exogenous pEGFP-Sumo1, ~135kDa). Only one band could be detected when membrane was probed with anti-Sumo1 antibody (Fig. S1Ab, lane 2, endogenous Sp1 Sumoylated with exogenous EGFP-Sumo1, ~135kDa). However, the samples loaded on SDS-Gel did not contain equal amounts of protein (Supplementary figures, (S1A and C), but, qualitatively, data showed that exogenous Sp1 was Sumoylated in LECs as reported previously by other investigators. To further assess the Sumoylated/deSumoylated status of Sp1, nuclear extract was used to perform Sp1 sandwich/Sumo1-ELISA (with anti-Sp1 monoclonal and polyclonal antibodies). Sumoylated Sp1 was subtracted from the total Sp1 amount, and is presented as deSumoylated Sp1 (gray bars). This experiment revealed that the level of the Sumoylated form of Sp1 in pEGFP-Sumo1 transfected LECs was four times higher than in pEGFP-Vector transfected LECs (Fig. S1B).

Because identifying which specific lysine (K) residue of Sp1 is Sumoylated requires mutagenesis, it was necessary to determine whether exogenous Sp1 is Sumoylated in LECs. We co-transfected SRA-hLECs with either pEGFP-Vector plus pCl-neo-HA-Sp1 or pEGFP-Sumo1 plus pCl-neo-HA-Sp1. Cells were processed for immunoprecipitation (IP) using anti-HA monoclonal antibody. Probing immunoprecipitates with anti-HA or anti-Sumo1 polyclonal antibodies revealed a discrete slower migrating band in monoclonal HA-IPs from extracts co-expressing pEGFP-Sumo1 plus pCl-neo-HA-Sp1 (Fig. S1Ca, upper panel, lane 2; pEGFP-Sumo1 + pCl-neo-HA-Sp1, approximately ~145kDa) recognized with anti-HA polyclonal and anti-Sumo1 polyclonal antibodies (Fig. S1Cb, upper panel, lane 2; pEGFP-Sumo1 + pCl-neo-HA-Sp1, approximately ~145kDa). For further validation of exogenous Sp1 Sumoylation, Sp1 sandwich/Sumo1-ELISA (with LECs transfected with pEGFP-Sumo1 + pCl-neo-HA-Sp1) showed ~50% reduction in deSumoylated Sp1 form (gray bars). Collectively, data indicated that Sp1 is exogenously Sumoylated.

**Figure 7:**
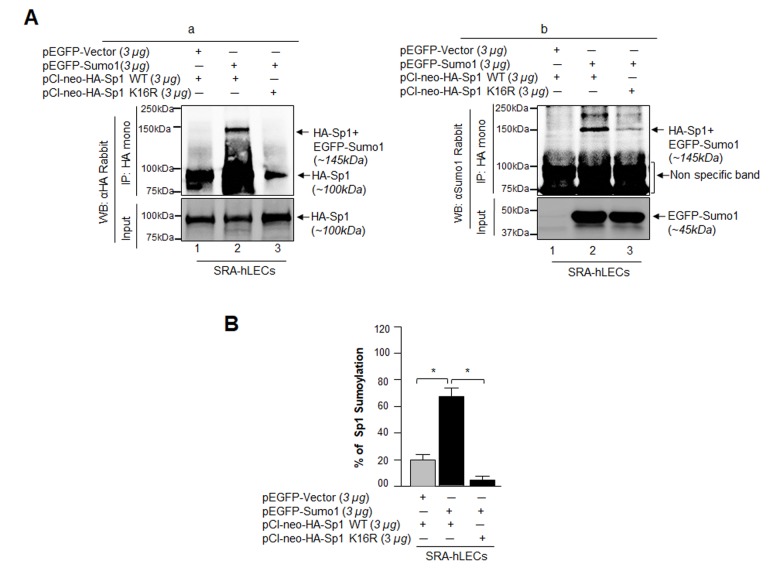
**Sumo1 modified Sp1 at K16 residue *in vivo*.** (**A**) SRA-hLECs (1.2X10^6^) were transfected with pEGFP-Sumo1 (3µg) along with Sp1 WT (3µg) or its mutant Sp1K16R (mutated at Sumoylation sites) using (3µg) plasmid as indicated. Exogenous Sp1 was immunoprecipitated (IP) from cell lysates containing equal amounts of proteins, and its Sumoylated form was detected with anti-HA (**Ba**) and anti-Sumo1 (**Bb**) rabbit polyclonal antibodies as indicated. Cell lysates were prepared and subjected to IP using anti-HA monoclonal antibody. IP with anti-HA monoclonal antibody shows a single-exogenous Sumoylated band at ~145 kDa (lane 2, pEGFP-Sumo1+pCl-neo-HA-Sp1WT). No Sumoylation band could be detected in cell extracts of pEGFP-Vector+pCl-neo-HA-Sp1WT or pEGFP-Sumo1+pCl-neo-HA-Sp1-K16R transfected cells (**B**, **a** and **b**; lanes 1 and 3). (**B**) SRA-hLECs were transfected with pCl-neo-HA-Sp1WT plus pEGFP-Vector, or pCl-neo-HA-Sp1WT plus pEGFP-Sumo1, or pCl-neo-HA-Sp1-K16R plus pEGFP-Sumo1. 48h later, total cell lysates were prepared and processed for Sumo1-ELISA assay according to the manufacturer’s protocol (EpiQuik^TM^) to measure the Sumoylated form of Sp1. Data represent mean ± SD from three independent experiments: pCl-neo-HA-Sp1WT plus pEGFP-Vector, vs pCl-neo-HA-Sp1WT plus pEGFP-Sumo1, vs pCl-neo-HA-Sp1 K16R plus pEGFP-Sumo1 (*p<0.001).

To ascertain if K16 is indeed the Sumoylation motif of Sp1 in LECs as reported for other cell types [23,32,34], we mutated K to arginine (R) (DEMTAVVRIEKGVGG) in full Sp1 cDNA (1-788aa) construct, (pCl-neo-HA-Sp1 WT) and generated Sp1 mutant K16R (pCl-neo-HA-Sp1-K16R). These plasmids were used to transfect LECs along with pEGFP-Sumo1 as indicated in Fig. 7. In vivo Sumoylation was conducted as described in Materials and Methods. As shown in Fig. 7B, a Sumoylated band of pCl-neo-HA-Sp1 WT plus EGFP-Sumo1 (Fig. 7B, a and b, ~145kDa, lane 2) was observed with both anti-HA and anti-Sumo1 polyclonal antibodies, while no Sumoylated protein band of pCl-neo-HA-Sp1-K16R plus pEGFP-Sumo1 was visible (Fig. 7B a and b, lane 3), confirming that Sp1-K16 is the major Sumoylation site in Sp1. Nonetheless, immunoblot analysis with Sumo1 antibody revealed non-specific proteins bands (75 and 100kDa), suggesting Sumo1 antibody cross-reacted to these protein bands. However, the bands were present in equal intensity in all three lanes, demonstrating equal loading. Next we performed Sp1-specific sandwich/Sumo1-ELISA assay using anti-HA or anti-Sumo1 antibody to validate our finding and to quantify the levels of Sumoylated and deSumoylated forms of exogenous Sp1. Data analyses revealed that EGFP-Vector plus pCl-neo-HA-Sp1 WT transfected LECs showed ~18% of Sp1 Sumoylation, which increased to 70% in pEGFP-Sumo1 plus pCl-neo-HA-Sp1 WT transfected LECs. However, no Sp1 Sumoylated form was detected in pEGFP-Sumo1 plus pCl-neo-HA-Sp1K16R transfected cells, suggesting that K16 residue is a major site of Sp1 Sumoylation. To avoid any artefactual effects, we performed similar *in vivo* experiments, using Sp1 deleted construct of Sp1 (pCl-neo-HA-Sp1 [1-293] WT or its mutant), as shown in Fig. S2. Data authenticated that indeed K16 residue is a Sumo1 conjugation site in Sp1 and overexpression of Sumo1 (free Sumo1 in cells) dramatically enhanced Sp1 Sumoylation. However, we could not detect any other Sp1 Sumoylation band as reported by Gong et al, 2014 [53].

### Sp1K16R mutated at Sumo1 site or Senp1 promoted Sp1-DNA binding, and overexpression of Sumo1 did not affect Sp1’s DNA binding potential

**Figure 8:**
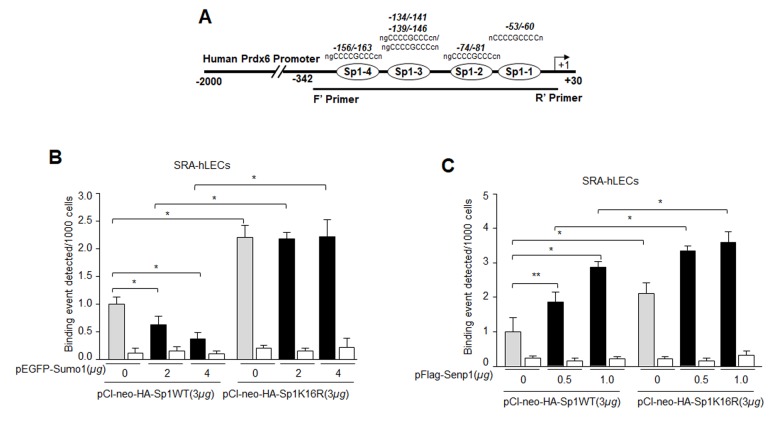
**Mutagenesis and *in vivo* DNA binding assay revealed increased binding of Sumoylation-deficient Sp1K16R to Sp1 site in Prdx6 promoter by skipping aberrant Sumoylation effect.** (**A**) Schematic representation of the regulatory region of proximal promoter of Prdx6 gene containing GC-box (Sp1 binding sites) showing primer location used in ChIP assay. (**B**) Sumo1 failed to affect Sp1-DNA binding activity in Sumoylation-deficient Sp1K16R transfected LECs. SRA-hLECs were transfected with either pCl-neo-HA-Sp1 or its mutant pCl-neo-HA-Sp1K16R alone or cotransfected with different concentrations of pEGFP-Sumo1. ChIP experiment was carried out as described in Materials and Methods. Chromatin samples prepared from LECs cotransfected with pEGFP-Sumo1 with either pCl-neo-HA-Sp1 or its mutant pCl-neo-HA-Sp1K16R were subjected to ChIP assay with a ChIP grade antibody, anti-HA (gray and black bars) and control IgG (open bars). The DNA fragments were used as templates for RT-qPCR by using primers designed to amplify −342 to +30 region of the *Prdx6* gene promoter bearing Sp1 binding sites as shown. Histogram shows the amplified DNA through real-time qPCR analysis. 0 µg vs 2 µg and 4µg pEGFP-Sumo1, pCl-neo-HA-Sp1 WT vs pCl-neo-HA-Sp1K16R (*p < 0.001). (**C**) Senp1 overexpression showed increased Sp1 DNA binding of Sp1WT and comparable to Sumoylation deficient Sp1K16R. SRA-hLECs were cotransfected with pFlag-Senp1 with either pCl-neo-HA-Sp1 or mutant pCl-neo-HA-Sp1K16R as indicated. ChIP assay was conducted as described above using anti-HA antibody. Histograms represent the concentration dependence of Senp1-induced enrichment of Sp1 at its binding sites in Prdx6 gene promoter. 0 µg vs 0.5 µg and 1µg pFlag-Senp1, pCl-neo-HA-Sp1 WT vs pCl-neo-HA-Sp1K16R (**p<0.05; *p < 0.001).

(Figs 1-6) suggest a possible role for oxidative stress and aberrant Sumoylation in dysfunction of Sp1-DNA binding activity. We posited that Sumo1-deficient Sp1K16R should have increased DNA binding activity, by escaping oxidative stress-induced aberrant Sumoylation. To confirm this, chromatin extracted from LECs co-expressing Sumo1 along with Sp1WT or its mutant (Sp1K16R) at Sumo1-motif (Fig. 8B) immunoprecipitated with IgG control or anti-HA monoclonal antibody was subjected to qPCR analysis using Prdx6 gene region specific primers denoted in Fig.3. Sumoylation-deficient Sp1 (Sp1K16R) showed significantly increased binding to its site present in Prdx6 gene in comparison to WT Sp1 (Fig. 8B). Sumo1 overexpression did not attenuate its DNA binding activity. (Fig. 8B, black bars). Similarly, we performed ChIP assay with deSumoylating agent Senp1 along with either Sp1WT or its mutant (Sp1K16R). As expected, LECs overexpressing Senp1 with WT Sp1 showed significantly higher Sp1-DNA binding activity, whereas Sp1K16R co-expression with Senp1 showed further increase in Sp1-DNA binding in comparison to Sp1 (Fig. 8C), indicating the presence of some minor Sumoylation sites in Sp1. However, data indicated that Sumoylation-deficient Sp1 or deSumoylation agent Senp1 enhanced the Sp1-DNA binding activity in *Prdx6* gene promoter, and suggested the involvement of Sumoylation in the loss of Sp1 activity.

### Mutation of Sp1K16R at Sumoylation site enhanced its transactivation potential by stabilizing its cellular availability

**Figure 9:**
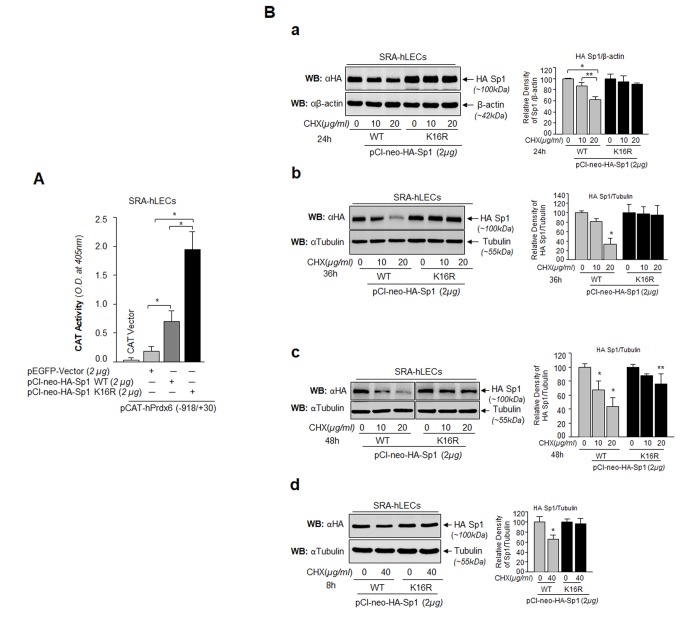
**Sp1 (K16R) mutated at Sumoylation site enhanced its transcription potential by increasing steady state of Sp1 in cells.** (**A**) SRA-hLECs were cotransfected with wild type Prdx6 promoter linked to CAT along with either pCl-neo-HA-Sp1 or pCl-neo-HA-Sp1K16R as shown. After 72h cell lysates were analyzed for CAT activity. Histograms represent values derived from three independent experiments. *p < 0.001. (**B**) Relative protein stability of Sp1WT vs Sumoylation-deficient mutant Sp1K16R. SRA-hLECs were transiently transfected with pCl-neo-HA-Sp1WT or its mutant, pCl-neo-HA-Sp1K16R. After 36h, the transfectants were treated with different concentrations of CHX (10 and 20µg/ml) for 24h (**Ba**) or CHX (10 and 20µg/ml) for 36h (**Bb**) or CHX (10 and 20µg/ml) for 48h (**Bc**) or CHX (40µg/ml) for 8h (**Bd**) or as indicated. Total lysates with equal amounts of proteins were western blotted (WB) with anti-HA antibody. Anti-β-actin or anti-Tubulin antibodies were used as loading control. The percentage of remaining Sp1 (Sp1WT and its mutant Sp1 K16R) protein after the CHX translational inhibitor treatment is presented as histogram in right side of Western blot based upon densitometry quantitation. Control vehicle (DMSO) vs CHX treated, **p<0.05; *p<0.001.

Transactivation assay was performed to determine if the increased binding of Sumo1-deficient Sp1 (Fig. 8B) was functional and provided more transactional activity in activating transcription of its target gene, Prdx6. As shown in Fig. 9A, hLECs co-transfected with Sumo1-deficient pClneo-HA-Sp1K16R (Fig. 9A, Black bars) along with Prdx6 promoter displayed significantly higher Prdx6 transcription compared to pClneo-HA-Sp1 WT (Fig. 9A, Dark gray bar). Next, we tested whether this enhanced transactivation potential was related to its abundant availability to its GC-box response element(s) in Prdx6 promoter due to Sp1’s increased stability. We analyzed the cellular stability of Sp1WT and its mutant, Sp1K16R at different time points as shown in Fig. 9B, by eliminating *de novo* protein synthesis with cycloheximide (CHX) treatment, a translational inhibitor. SRA-hLECs transiently transfected with pCl-neo-HA-Sp1WT or its mutant (pCl-neo-HA-Sp1K16R) were treated with CHX as indicated in Fig. 9B. We observed that Sumo1 deficient Sp1K16R was more stable than the Sp1WT; the remaining protein of Sp1WT and its mutant forms after treatment with CHX is also shown in the histogram (percentages) right side of the protein bands based on densitometry quantitation analysis. An analysis of data revealed that cellular abundance of mutant K16R proteins was significantly higher than that of Sp1WT protein with 10 μg/ml and 20 μg/ml treatment of CHX for 24h, 36h and 48h (Fig. 9Ba, Bb and Bc), suggesting that Sumoylation destabilized Sp1 by mediating its degradation [23,34,53]. We also observed that at a higher concentration of CHX (40μg/ml) for 8h, Sp1K16R mutated at Sumo1 site (Fig. 9Bd), showing greater stability compared to Sp1 WT. Similar results were obtained with mLECs (data not shown). We conclude that the decline in Sp1 abundance in cells might be due to changes in Sumoylated Sp1 stability (Fig. 9).

### Sp1K16 R gained potential for protecting cells from oxidative and aberrant Sumoylation stresses

**Figure 10:**
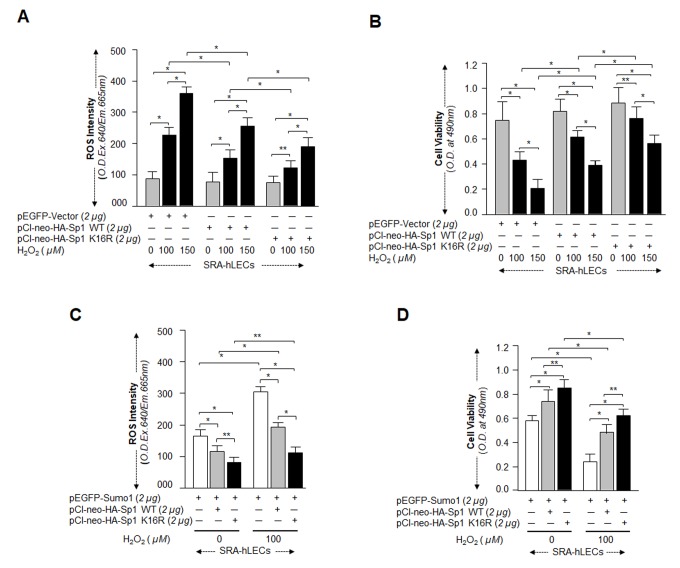
**Sp1K16R mutated at Sumo1 binding site provided enhanced cytoprotection against oxidative stress.** (**A** and **B**) SRA-hLECs were transfected with either pEGFP-Vector, pCl-neo-HA-Sp1, or pCl-neo-HA-Sp1K16R and then exposed to different concentrations of H_2_O_2_ as indicated. After 8h of H_2_O_2_ exposure, ROS intensity was quantified with CellROX deep red reagent (**A**). 24h later viability of cells was analyzed by MTS assay (**B**) as shown. Histogram values represent mean ± SD of three independent experiments. 0 vs 100 vs 150µM H_2_O_2_ and pEGFP-Vector vs pCl-neo-HA-Sp1 WT vs pCl-neo-HA-Sp1K16R (**p<0.05; *p<0.001). (**C** and **D**) SRA-hLECs were transfected with pEGFP-Sumo1 along with either pCMV-Vector (open bars), pCl-neo-HA-Sp1 (gray bars), or pCl-neo-HA-Sp1K16R (black bars), and then exposed to oxidative stress. ROS intensity (**C**) and cell viability (**D**) are presented as histograms. Values represent mean ± SD of three independent experiments. 0 vs 100µM H_2_O_2_ and pEGFP-Sumo1 vs pEGFP-Sumo1 plus pCl-neo-HA-Sp1 WT vs pEGFP-Sumo1 plus pCl-neo-HA-Sp1K16R (**p<0.05; *p<0.001). Sumoylation-deficient Sp1K16R (black bars) showed significantly higher protection and reduced ROS production, indicating that mutant Sp1K16R was more effective in protecting cells from oxidative stress Sumoylation-mediated insults.

Next, we asked whether Sumoylation-deficient Sp1K16R had increased capacity to protect cells against oxidative or aberrant Sumoylation-mediated stresses. SRA-hLECs overexpressing pCl-neo-HA-Sp1WT or pCl-neo-HA-Sp1K16R were exposed to different concentrations of H_2_O_2_ as indicated in Fig. 10A and 10B. Quantitation of ROS by CellROX Deep Red dye and cell viability by MTS assay revealed that pCl-neo-HA-Sp1K16R transfected cells showed significantly reduced ROS (Fig. 10A) and increased viability (Fig. 10B) compared to pCl-neo-HA-Sp1WT. We measured the synergistic effect of Sumo1 and oxidative stress on protective efficacy of Sp1K16R, as we assumed that this condition might occur *in vivo* in aging or aged cells. The experiments were similar to those described above, but used cells overexpressing Sumo1. When assayed for ROS and cell viability, the transfectants bearing Sp1K16R were found to be highly efficient in reducing ROS (Fig. 10C), and engendered more resistance against oxidative and Sumo1-induced insults (Fig. 10D). Collectively, data suggest that Sp1K16R rescued the cells by blunting oxidative and increased Sumoylation mediated stresses.

### Sumoylation-deficient Prdx6K122/142R fused to transduction domain (TAT) internalized in cells and lessened oxidative stress and its induced aberrant Sp1 Sumoylation

**Figure 11:**
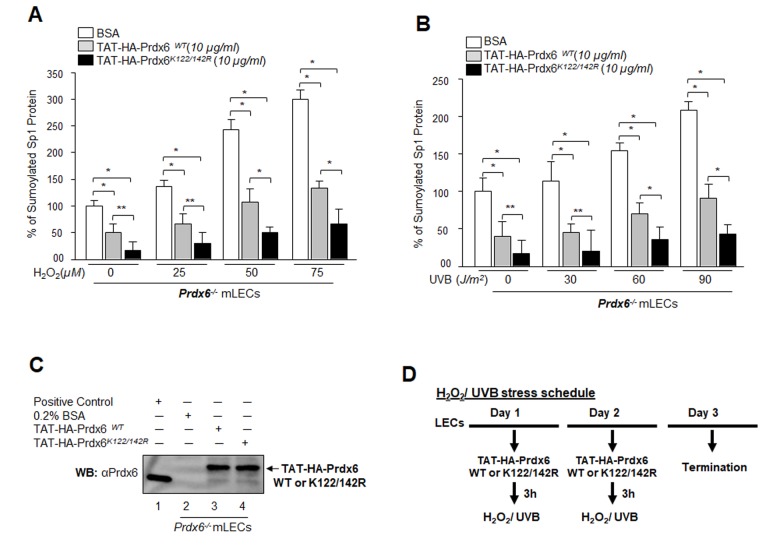
**Sumoylation-deficient Prdx6K122/142R fused to transduction protein domain (TAT) internalized in cells and blunted oxidative stress-induced aberrant Sumoylation.** (**A** and **B**) *Prdx6^-/-^* mLECs were transduced with Sumoylation-deficient protein, TAT-HA-Prdx6K122/142R conferred higher resistance to oxidative stress-induced Sumoylation than did Prdx6WT. *Prdx6^-/-^* LECs were pretreated with TAT-HA-Prdx6 WT or TAT-HA-Prdx6K122/142R and then exposed to different concentrations of H_2_O_2_ (0, 25, 50 and 75µM) and/or UVB (0, 30, 60 and 90J/m^2^). 48h later, nuclear extracts containing equal amounts of proteins were processed for Sumo1-ELISA assay to assess the relative levels of Sp1 Sumoylation in Prdx6 WT (gray bars) and its mutant Prdx6K122/142R (black bars) transduced in cells as shown. Data represent the mean ± SD from three independent experiments (**p<0.05, *p<0.001). (**C**) Transduction of TAT-HA-Prdx6 and TAT-HA-Prdx6K122/142R into cells. An aliquot of 10 μg/ml recombinant protein was added to culture media and transduction of TAT-HA-Prdx6 (Lane 3) and TAT-HA-Prdx6K122/142 R (Lane 4) was assessed using WB by anti-Prdx6 antibody. (**D**) Represents the TAT-HA-Prdx6 and TAT-HA-Prdx6K122/142R following H_2_O_2_ and/or UVB oxidative exposure treatment schedule.

Results from the current study as well as mounting evidence from other reports including our own indicate that oxidative stress is a primary culprit in malfunction or pathobiology of cells and tissues. Conceivably, a logical strategy for overcoming the adverse processes might be reducing intracellular ROS-driven oxidative stress and Sumoylation signaling by means of naturally occurring antioxidants such as Prdx6 K122/142R mutated at Sumo1 site [12]. We previously demonstrated that Prdx6 is Sumoylated at K122 and K142, and Sumoylation of Prdx6 reduces its protective potential, while mutant Prdx6 at K122/142R gains significant protective potential and escapes oxidative stress-induced aberrant Sumoylation signaling. To test whether Prdx6K122/142R protects cells against oxidative stress-induced deleterious signaling, we delivered Sumoylation-deficient Prdx6 linked with TAT transduction domain (TAT-HA-Prdx6K122/142R) [5,12] to Prdx6-deficient LECs subjected to oxidative stresses. As shown in Fig. 11, oxidative stress induced by H_2_O_2_ (Fig.11A) or UVB (Fig.11B) significantly enhanced the Sp1 Sumoylation. Intriguingly, that increase in Sumoylation was significantly blunted in *Prdx6^-/-^* LECs treated with mutant Prdx6 at K122/142R (Fig. 11A, open bars vs gray bars vs black bars) or UVB (Fig. 11B, open bars vs gray bars vs black bars) compared to TAT-HA-Prdx6WT as evidenced through Sp1/Sumo1-ELISA assay. These findings suggest that Sumoylation-deficient Prdx6 may efficiently protect cells by rescuing Sp1 from deleterious signaling evoked by oxidative stress and aberrant Sumoylation.

### Delivery of TAT-HA-Prdx6K122/142R increased Sp1-DNA binding to Prdx6 promoter in LECs under oxidative stress and aging hLECs

**Figure 12:**
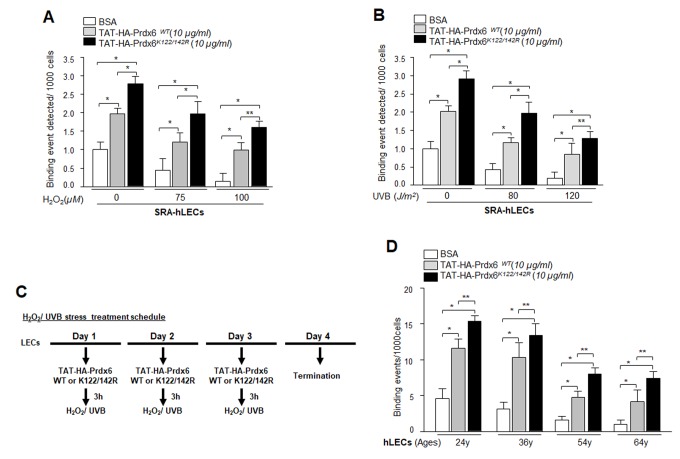
**Enhanced Sp1 binding to Prdx6 promoter in cells transduced with Sumoylation–deficient Prdx6 against oxidative stress.** (**A** and **B**) SRA-hLECs were transduced with TAT-HA-Prdx6WT and its mutant TAT-HA-Prdx6K122/142R mutated at Sumoylation sites recombinant protein followed by different concentrations of H_2_O_2_ (**A**) or UVB (**B**) exposure as indicated. ChIP assay was carried out using ChIP grade anti-Sp1 antibody. The DNA fragments were used as templates for qPCR by using primer designed to amplify -342 to +30 region of the human Prdx6 promoter bearing GC-box (Sp1 sites). Histogram shows the amplified DNA with real-time PCR analysis; open bars vs gray bars vs black bars. The data represent mean ± SD from two independent experiments (**p<0.05; *p<0.001). (**C**) The H_2_O_2_ and/or UVB treatment schedule. (**D**) *In vivo* DNA binding assay revealed that transduction of Prdx6 and its mutant at K122/142R linked to TAT reactivated binding activity of Sp1 in aging primary hLECs. Primary hLECs of variable ages were transduced with TAT-HA-Prdx6 WT or its mutant TAT-HA-Prdx6K122/142R. ChIP experimentation was conducted using anti-Sp1 antibody. Immunoprecipitated DNA fragments were purified and processed for qPCR analysis using specific primers. Histograms represent the TAT-HA-Prdx6 WT and its mutant-induced enrichment of Sp1 at GC-box (Sp1 binding sites) in Prdx6 gene promoter. Open vs gray and black bars, and gray vs black bar; **p<0.05, *p < 0.001. Data revealed a significant augmentation of Sp1 binding by TAT-HA-Prdx6K122/142R in all ages of LECs, but younger cells were more responsive.

Previous experiments in this study showed that expression of Prdx6 in LECs reduced the Sp1 Sumoylation caused by oxidative stress and protected those cells. We next asked whether delivery of Prdx6 linked to TAT transduction domain protected cells by augmenting the Sp1-DNA binding mechanism. Oxidative stress and its induced adverse signaling has been shown to dysregulate transcriptional activity by damaging DNA binding activity and reducing expression of antioxidants, and antioxidant application is known to reverse the process [3,10,54–56]. We performed ChIP assay using anti-Sp1 antibody. SRA-hLECs or aging LECs transduced with TAT-HA-Prdx6 WT and its mutant at K122/142R were subjected to oxidative stressors as described in Materials and Methods (Fig. 12). Chromatin samples prepared from cells were immunoprecipitated with anti-Sp1 antibody, and IP samples were analyzed with qPCR by using specific primers (Fig. 3A) designed for ChIP assay. As shown in Figure 12, cells pretreated with TAT-HA-Prdx6 WT (Fig. 12, gray bars) had significantly higher Sp1-DNA binding both with and without H_2_O_2_ or UVB in comparison to untreated LECs (Open bars). On the other hand, we found significantly higher enrichment of Sp1 in LECs transduced with Sumoylation-deficient (Prdx6K122/142R, Black bars) in comparison to untreated (Open bars) and even WT Prxd6 (gray bars). Younger cells were more responsive than aged cells. As a whole, data demonstrated that, during aging and oxidative stress, application of Prdx6 in the form of Sumoylation-deficient TAT-Prdx6K122/142R is deliverable, internalizes in cells (Fig. 11C), and can protect cells by restoring DNA binding of Sp1.

## DISCUSSION

Sumo modification is a critical regulator of many types of proteins, including transcription factors. Aberrant Sumoylation caused by oxidative stress disrupts genetically allotted functions of proteins, and lead to etiopathology and progression of many human diseases, including age-associated disorders [28–31,57]. Most Sumoylated transcription factors, including Sp1, have been shown to repress gene transcription, and augmentation of this process during aging or oxidative stress results in death signaling [11,39,43,58–62]. Sp1, a ubiquitous transcription factor, activates many genes by binding to GC-rich sequences present mostly in TATA-less promoter, which is crucial for the basal transcription of these genes [9,11,21,49,63]. However, mounting evidence indicates that Sp1 can act as a regulator of transcription of various genes [9,11,64,65] including both prosurvival and proapoptotic genes [9,11,64–67].

Sp1 expression can be affected by various stimuli including oxidative stress and aging as well as via its interaction with other proteins [9,49,66]. Its activity can be highly modulated by various posttranslational modifications, such as Sumoylation, phosphorylation, glycosylation, acetylation and oxidation [27,34,68]. In the present study, we found that levels of Sp1 mRNA and protein as well as expression of its target gene, antioxidant protein Prdx6, declined with increases in ROS-driven oxidative load in aging LECs. The decline was linked to reduction of Sp1's DNA binding efficiency as well as reduced cellular abundance (Fig.1), indicating that repression of Prdx6 is at transcriptional levels. It is justifiable to mention that there was no disruption of transcriptional machinery during aging as there was no change in internal control (internal control gene or protein expression). We previously showed that aging hLECs, *Prdx6^-/-^* (a model for aging) and aged trabecular meshwork (TM) cells bear significantly higher ROS expression levels than cells of younger age [10,16,69]. Ammendola reported that Sp1 binding is dysregulated in aging due to changes in redox environment with no altered expression of other molecules examined [54]. Moreover, this age-related adverse phenomenon is not limited to Sp1; several reports show that activity of transcription factors, such as Nrf2, declines with age and the decline is correctable [10,56]. Furthermore, aging and oxidative stress can directly modulate the induction of several genes encoding transcription factors, including Fos, Jun, Myc, Erg1, NF-кB, HSF1 [56,70–73]. In the current study we also extended our previous finding(s) [5,12] to explore causes of the observed dysregulation of Sp1 and repression of Prdx6 gene during oxidative stress. We observed that Sp1-DNA binding was reduced to its response element(s) present in both mouse and human Prdx6 gene promoter in cells exposed to oxidative stress, and that the binding capacity was directly connected to intracellular oxidative load and reduction in Prdx6 expression (Fig. 2). ROS-evoked oxidative stress can modulate expression levels and DNA binding efficiency of many transcription factors that may lead to reduced or increased expression of their target genes [27,74–76]. We observed that reduced Sp1 binding to Prdx6 promoter was connected to reduced expression of Prdx6 mRNA (without affecting internal control gene), implicating that transcriptional machinery is not influenced during oxidative stress. Nonetheless, we recognize that it is difficult to give a general statement about oxidative and aging stress-evoked deleterious signaling-mediated cell damage as they initiate multiple signaling. We believe that this requires further study to discern the effects of aging or oxidative stress on epigenetic/genetic levels.

Oxidative-induced injurious signaling is known to be a prime cause of cellular insult during aging [77]. In humans, oxidative stress-induced increased ROS along with reduced antioxidant defense capacity is now believed to cause many age-related degenerative disorders, including cataractogenesis, neurodegenerative disorders, glaucoma, macular degeneration, cancer, diabetes and cardiovascular disease [2,5,12,31,69,78]. Intracellular ROS is tightly regulated by antioxidant defense proteins, to maintain correct physiological levels of ROS. Dysregulation of transactivators of antioxidant defense genes can lead to reduced expression of antioxidant protein, which may adversely affect physiological levels of ROS. Thus genetically allotted physiological control of the transcriptional machinery of defense genes, including Prdx6, is of utmost importance.

Oxidative stress has been reported to accelerate/decelerate Sumo1 conjugation to proteins via direct or indirect mechanisms, thereby modifying their functions [5,40,43,63,79–81]. In the current study we found that Sp1 was endogenously Sumoylated and its Sumoylation status was dramatically increased with an increase of oxidative stress, as shown in Sumoylation experiments with Sp1 (Fig. 4). The aberrant Sp1 Sumoylation was linked to increased Sumo1 expression and reduced expression of Senp1. An excess of oxidative stress has been shown to aberrantly modulate Sumoylation process by increasing or decreasing proteins Sumoylation and their function(s) [5,12,82–84]. This can happen due to inactivation of Sumo isopeptidases either by dimerization or overoxidation of their catalytic cysteine [5,23]. Our results revealed increased Sumoylation of Sp1 with increased oxidative stress and Sumo1 expression, which possibly occurred due to reduced expression and inactivation of Senp1, a deSumoylating enzyme for Sumo1 conjugation (Fig. 4), during aging, arguing that gene regulatory transcriptional state is active in aging and the condition can be remedied (as observed in Figs. 11 and 12). Other possibilities may exist: (i) increased expression of Sumo1 during oxidative stress that can result in increased abundance of free Sumo1 in cells ([5] and current study, Fig. 5), and (ii) ROS directly or indirectly affecting Sumo E3 ligase activity leading to increased conjugation [79,85]. Alternatively, because oxidative stress modifies the phosphorylation status of proteins including Sp1, it may in turn modify Sumoylation of Sp1. Among several specific substrates for Sumos that have been found to be affected in response to oxidative stress are Sp1, NF-кB, LEDGF, HIPK2, TP53INP1 and Prdx6 [5,12,23,34,60,83,86–88]. Moreover, in studying the molecular effect of oxidative-induced aberrant Sumoylation of Sp1 on Sp1-DNA binding activity, both *in vitro* and *in vivo* DNA-binding experiments with Sp1 to its GC-response element in Prdx6 promoter revealed significant dysregulation of Sp1-binding in cells overexpressing Sumo1. In contrast, cells overexpressed with Senp1 displayed increased Sp1-binding. This reduction in Sp1 binding indicates an involvement of Sumo1 that plays negatively in Sp1-DNA binding activity as shown in Fig. 6. The data argue that the repression of Prdx6 in cells may be a cause of reduced Sp1 DNA binding to its response element in Prdx6 promoter in response to oxidative-induced increased Sumoylation signaling that influences Sp1 cellular abundance and thereby reduces enrichment to its binding sites. The reduction in Sp1-DNA interaction appears to have two possible causes: (i) Sp1 became a target for quick degradation due to aberrant Sumoylation [32,79,83] and Fig. 9B, or (ii) Sp1 binding was inactivated by oxidative stress (Figs. 1, 2, 3 and [3,54]). However, real time expression analysis and DNA-binding assay using nuclear extracts containing equal amounts of Sp1 protein (Figs. 1) revealed that both the reduced expression and reduced DNA binding efficiency of Sp1 was involved in dysregulation of Sp1 activity in response to aging or oxidative stress and oxidative stress-induced aberrant Sumoylation signaling (Figs. 5 and 6).

Furthermore, if oxidative stress-induced aberrant Sumoylation of Sp1 plays a role in dysregulation of Sp1, it is conceivable that Sumo1-deficient Sp1 would bear transactivation activity in activation of its target gene Prdx6 during oxidative stress. We recognize that a Sumo1 binding site in Sp1 has been reported. Nevertheless, as posttranslational modification of protein can vary with the cell type and cellular microenvironment, we examined whether Sp1 is Sumoylated in LECs *in vivo*. Mutagenesis along with *in vivo* Sumoylation experimentation revealed Sumo1 mediated-Sumoylation of Sp1 at K16 residue (Fig. 7 and Supplementary Fig. S1 and S2), a finding consistent with reports by others [23,32,34]. We called this construct Sumo1-deficient Sp1K16R and tested its transactivation potential. Fig. 8 shows that DNA binding activity of Sumo1-deficient Sp1 significantly increased in Prdx6 promoter even in cells overexpressing Sumo1. In contrast, Sp1WT activity was dramatically attenuated, indicating the role of Sumoylation in reduction of Sp1 activity. The data were further supported when cells overexpressing Senp1 showed restoration of the DNA-binding activity of Sp1 (Fig. 8B). While testing the functionality of their DNA-binding activity in transactivation experiments, we found that Sumoylation-deficient Sp1 had greater transactivation activity than its counterpart, Sp1WT (Fig. 9A). This elevated activation potential of Sumo1-deficient Sp1 was connected with its enhanced cellular abundance due to increased stability as evidenced by cellular stability assay (Fig. 9B). One of the most salient observations of the present study was that LECs overexpressing Sumoylation-deficient Sp1 gained resistance against oxidative stress and aberrant Sumoylation-mediated injurious signaling, and those cells survived better (Fig. 10). The improved condition of the Sumoylation-deficient Sp1 was due to its escaping the aberrant Sumoylation processes and thereby activating Prdx6 ([5,9] and Fig. 9). Posttranslational modification of Sp1 has been shown to influence its transcription activity and stability [12,25,26,34,35]. Glycosylation of Sp1 can stimulate or repress DNA binding and transcription [89]. In basal condition Sp1 is Sumoylated at N-terminal, which negatively regulates Sp1 transcription activity [32]. However, Sumo1 conjugation of Sp1 has been reported to enhance its activity [53]. This discrepancy may be related to experimental conditions. Our results and other reports clearly show that Sumoylation of Sp1 leads to its dysregulation [11,32,35,90]. Furthermore, H_2_O_2_ has been shown to decrease NOS-3 promoter activity by reducing the binding capacity of Sp1 [91].

It was intriguing to observe that the level of Sp1 Sumoylation was significantly higher in redox active *Prdx6^-/-^* cells and in aging/aged cells, and that these were much more susceptible to oxidative stress than were *Prdx6^+/+^* cells (Fig. 4) and younger hLECs (data not shown). This suggests a pivotal role for Prdx6 in abating oxidative stress-mediated aberrant Sumoylation signaling, at least in eye lens. Previous studies [5,12,43], as well as the current research show that oxidative stress and oxidative stress-induced overstimulation of Sumoylation pathways are a major culprit in initiating etiopathobiology of cells or cell injuries. We think that damaging oxidative load in aging cells or cells in redox-active stage can be remedied by enhancing the natural defense via providing an extrinsic supply of antioxidant, like Prdx6, capable of optimizing oxidative load [5,9,12]. Indeed, we found that delivery of Prdx6 reduced the UVB and H_2_O_2_-induced Sp1 Sumoylation in *Prdx6^-/-^* LECs (Fig. 11), restored Sp1-DNA binding activity (Fig. 12), and reactivated Sp1-DNA binding to Prdx6 promoter in aging hLECs (Fig. 12C). These data indicate the potential for Sumoylation-deficient Prdx6 [12] to be used as a therapeutic molecule to block oxidative-induced aberrant Sumoylation-driven pathogenic signaling.

In conclusion, we have described a novel mechanism—oxidative-evoked aberrant Sumoylation signaling—which dysregulates Sp1 and its target survival genes such as Prdx6 in eye lens cells affected by aging or oxidative stress. Taken together, findings of this study show that oxidative stress-evoked aberrant Sumoylation signaling attenuates Sp1 transactivation ability by aberrant Sumoylation of Sp1; lessening cellular stability and availability of Sp1 to GC-response elements in the target genes. Additionally, we found that Sumoylation-deficient Sp1 resisted aberrant Sumoylation processes induced by oxidative stress, further demonstrating the prime role of oxidative stress and its associated aberrant Sumoylation-mediated Sp1 dysregulation. Because adverse signaling is driven by oxidative stress and reduction in antioxidants, application of Sumoylation-deficient Prdx6 [12] may be considered to block or delay the oxidative and aberrant Sumoylation-mediated injurious signaling by reactivating survival transcription factors like Sp1.

## MATERIALS AND METHODS

### Cell culture

Human LECs used were of two types: (i) a cell line (SRA01/04) immortalized with SV40, and (ii) primary human LECs isolated from deceased persons of different ages. To avoid confusion, the remaining text will designate the immortalized LECs as SRA-hLECs, and the primary human (h) LECs as primary hLECs or hLECs.

The SRA-hLECs were derived from 12 infants who underwent surgery for retinopathy of prematurity [92] (a kind gift of Dr. Venkat N. Reddy, Eye Research Institute, Oakland University, Rochester, MI, U.S.A). These cells were maintained in DMEM with 15% FBS, 100µg/ml streptomycin, and 100µg/ml penicillin in 5% CO_2_ environment at 37°C as described previously [5,93].

### Isolation and generation of hLECs

Primary hLECs were isolated from normal eye lenses of deceased persons or healthy donors of different ages (16, 18, 21, 24, 26, 30, 52, 54, 56, 58, 62, 64, 65, 66, 74, 75, 75 and 76y) obtained from the Lions Eye Bank, Nebraska Medical Center, Omaha, NE, and National Development & Research Institute (NDRI), Inc., PA. According to regulation HHS45CFR 46.102(f), studies involving material from deceased individuals are not considered human subject research as defined at 45CFR46.102(f) 10(2) and do not require IRB oversight. For RNA expression and DNA interaction studies, lenses used from each group for this purpose were those aged 18, 24, 30, 56, 66 and 76y. The remaining lenses were used for generation of LECs for other experiments mentioned in this study. Briefly, the capsule was trimmed before explanting in 35mm culture dishes precoated with collagen IV containing a minimum amount of DMEM containing 15-20% fetal bovine serum (FBS), with a brief modification [16,94–96]. Capsules were spread by forceps with cell layers upward on the surface of plastic petri dishes. Culture explants were trypsinized and re-cultured. Cell cultures attaining 90 to 100 percent confluence were trypsinized and used for experiments [11,93,97]. Western analysis was used to validate the presence of αA-crystallin, a specific marker for LEC identity (data not shown). For the experiments, SRA-hLECs and/or hLECs were cultured in 96, 24, 48, 6 well plates, or 60 and 100 mm petri dishes according to the specific requirements of each experiment.

### Quantitation of intracellular ROS level by H2-DCF-DA and CellROX deep red reagent

Intracellular ROS level was measured by use of fluorescent dye dichlorofluorescin diacetate (H_2_DCFDA), a nonpolar compound that is converted into a polar derivative (dichlorofluorescein) by cellular esterase after incorporation into cells [16]. On the day of the experiment, the medium was replaced with Hank’s solution containing 10 µM H_2_DCFDA dye and cells were incubated. Following 30 min later, intracellular fluorescence was detected with excitation at 485 nm and emission at 530 nm by a Spectra Max Gemini EM (Mol. Devices, Sunnyvale, CA, USA).

ROS level were measure according to the company’s protocol (CellROX Deep Red Oxidative Stress Reagent, Catalog No. C10422, Thermo Scientific, Carlsbad, CA, USA). In brief, LECs (5 × 10^3^) transfected with pEGFP-Vector or pEGFP-Sumo1 with pCl-neo-HA-Sp1 or pCl-neo-HA-Sp1K16R cultured in 96-well plate, 48 h later cells were exposed with different concentration of H_2_O_2_. After 8h, CellROX deep red reagent was added with final concentration of 5*μ*M and cells were incubated at 37°C for 30 min. Media containing CellROX deep red reagent were removed and fixed with 3.7% formaldehyde. After 15 min, fluorescence signal were measured at Ex640 nm/ Em665 nm [12].

### Real-Time Reverse Transcriptase-Polymerase Chain Reaction (RT-PCR)

Total RNA from the primary hLECs directly detached from lenses (to avoid cell culture effect) was isolated using the single-step guanidine thiocyanate/phenol/chloroform extraction method (Trizol Reagent, Invitrogen). To examine the levels of Sp1, Prdx6, Senp1 and Sumo1, 0.5 to 2 micrograms of total RNA was converted to cDNA using Superscript II RNAase H-reverse-transcriptase. Quantitative real-time PCR was performed with SYBR Green Master Mix (Roche Diagnostic Corporation, Indianapolis, IN) in a Roche® LC480 Sequence detector system (Roche Diagnostic Corporation). PCR conditions of 10 min hot start at 95 ºC, followed by 45 cycles of 10sec at 95 ºC, 30 sec at 60 ºC and 10 sec at 72 ºC. The primer Sequence was: Prdx6 (Human), Forward: 5′-GCATCCGTTTCCACGACT -3′ and Reverse: 5′-TGCACACTGGGGTAAAGTCC-3′; Sp1 (Human), Forward: 5′-CCTGGATGAGGCACTTCTGT-3′ and Reverse: 5′-GCCTGGGCTTCAAGGATT-3′; Sumo1 (Human), Forward: 5′-AAGCCACCGTCATCATGTCT-3′ and Reverse: 5′-TTATCCCCCAAGTCCTCAGTT-3′; Senp1 (Human), Forward: 5′-TTCCTCGCTGATGACAACTG-3′ and Reverse: 5′-AGTGAGTCCATAAGTAGGATACAAGGT-3′; β-actin (Human), Forward: 5′-CCAACCGCGAGAAGATGA-3′ and Reverse: 5′-CCAGAGGCGTACAGGGATAG-3′. The relative quantity of the mRNA was obtained using the comparative CT method. The expression levels of target genes were normalized to the levels of β-actin as an endogenous control in each group.

### Transcription factor activation assay

Sp1 activation assay was performed according to manufacturer’s protocol (TransAM Sp1 Transcription Factor Assay Kit, Cat No 41296, Active motif, Carlsland, California, USA). In brief, 10µg of nuclear extract (up to 10µl diluted with complete lysis buffer) prepared from aging hLECs added to the strips well, following the addition of 40µl complete binding buffer contains 20pmol of the wild-type and/or mutated consensus oligonucleotide to each sample well. For blank well, 10µl of complete lysis buffer were used. The plate was incubated for 1h at room temperature (RT) with mild agitation. 100µl primary antibody (1:1000 in 1X antibody binding buffer) added after 3 wash with 1X washing buffer and incubated at RT for 1h without agitation. 100µl of diluted anti-rabbit HRP-conjugated antibody (1:1000 dilution in 1X antibody binding buffer) after three washing was added and incubated for 1h at RT. 100µl of developing solution was added to wells after four washing and incubated at RT in dark for 2 to 10 min. Finally, by addition of 100µl of stop solution, OD was recorded at 450nm.

### Sensitive Sp1 sandwich/Sumo1-ELISA

A total Sp1 protein and its Sumoylated form was measured through sandwich-ELISA (Abnova, Taipei City, Taiwan) and the EpiQuik *in vivo* universal protein Sumoylation assay kit in accordance with the manufacturer's instructions [5,12]. Briefly, in the sandwich-ELISA assay, total cell lysates and/or nuclear extracts were prepared from aging/aged hLECs, Prdx6*^+/+^*, *Prdx6^-/-^* (H_2_O_2_ and/or UVB exposed) and SRA-hLECs (transfected with different plasmid constructs as indicated in figures). Equal amounts of protein were loaded in an ELISA plate well coated with anti-Sp1(PAB6826, PAB18222, Abnova and ab59267, Abcam) / anti-Prdx6 (sc-101522, Santa Cruz and LF-PA0011, Ab Frontier, South Korea) / anti-HA (ab9110, Abcam) polyclonal antibody followed by incubation with anti-Sp1(LS-B6148, LS Bio), anti-Prdx6 (LF-MA0018, Ab Frontier, South Korea) or anti-HA (H9658, Sigma-Aldrich) monoclonal serum. After incubation with goat anti-mouse-HRP(sc-2354, Santa Cruz) conjugated secondary serum, OPD substrate solution was added for color development and optical density (OD)_490_ was monitored as described in our published protocol [5,12].

As noted above, the same cell extracts from transfectants or controls were used and Sumoylated Sp1 or Prdx6 or HA-Sp1 was detected by ELISA using an EpiQuik *in vivo* universal protein Sumoylation assay kit (Epigentek, Farmingdale, NY, USA). Equal amounts of proteins from the total or nuclear extracts were added to the strip wells, which were precoated overnight with either anti-Sp1 or anti-Prdx6 or anti-HA serum. They were then incubated in blocking buffer for 45 min, washed three times and incubated with Sumo assay buffer for 1h at room temperature. After three washes, Sumo1 antibody was added and the proteins were incubated for 15 min at room temperature. Subsequent to color development by a Sumo detection system, OD_450_nm was measured an ELISA plate reader. To obtain deSumoylated Sp1 or Prdx6 or HA-Sp1, we calculated total and Sumoylated Sp1 or Prdx6 or HA-Sp1 protein and subtracted the Sumoylated Sp1 or Prdx6 or HASp1 protein from total Prdx6 protein.

### Generation and validation of LECs isolated from lenses of *Prdx6^−/−^* and *Prdx6^+/+^* mice

All animal experiments followed the recommendations set forth in the “Statement for the Use of Animals in Ophthalmic and Visual Research” by the Association for Research in Vision and Ophthalmology (ARVO). The University of Nebraska Medical Center (UNMC) approved animal studies. LECs isolated from Prdx6-targeted mutants (*Prdx6^−/−^*) and wild type (*Prdx6^+/+^*) mice were generated and maintained in Dulbecco's modified Eagle's medium (DMEM) with 10% fetal bovine serum (FBS), as described earlier [33]. We used *Prdx6^−/−^* mutant and *Prdx6^+/+^* C57/B6, mice of the same sex and age. All animals were maintained under specific pathogen-free conditions in an animal facility. LECs were isolated from mice of identical age, and Western analysis was carried out to confirm the presence of αA-crystallin, a specific marker of LECs.

### Chromatin Immunoprecipitation (ChIP) assay

ChIP was performed using the ChIP-IT® Express (Cat. No. 53008; Active Motif, Carlsbad, CA, USA) and ChIP-IT® qPCR analysis kit (Cat. No. 53029; Active Motif, Carlsbad, CA, USA) following the manufacturer's protocol [11]. The following antibodies were used: control IgG and antibody specific to Sp1 (ab13370, Abcam) and/or HA (ab9110 and ab18181, Abcam). Real-time PCR or real-time quantitative PCR (qPCR) amplification was carried out using 5μl of DNA sample with primers [mouse promoter bearing Sp1 sites, forward primer: 5’-CGCAATTCTCGGTCTTGCGCTTC-3’ and reverse primer: 5’-GTGGTGACGCTGAGAACAAGGA-3’,positions -208/+27; and contiguous sequence to which Sp1 does not binds, forward primer: 5’-CCTGGTTCCTTACATATAAGGC-3’ and reverse primer: 5’-cctggtatagtatatgtccctg-3’, positions -2356/-2229 relative to the A in the ATG translation initiation codon, and human promoter within Sp1 binding sites, forward primer: 5’-catcacgtgtgcagagacggc-3’ and reverse primer: 5’-cacgtccccgagaagcagac-3’ ,positions -342/+30 relative to the A in the ATG translation initiation codon] specific to the Prdx6 promoter. The program for quantification amplification was 3min 94°C, 20s at 95°C, 30s at 59°C and 30s at 72°C for 36 cycles in 25μl reaction volume (RT-PCR) or 2min at 95°C, 15s at 95°C, 20s at 58°C and 20s at 72°C for 40 cycles in 20μl reaction volume (RT-qPCR). Data obtained with RT-PCR run on 1% agarose gel and visualized band under UV and image captured or Data obtained with qPCR were plotted and presented in the form of histogram.

### Construction of human Prdx6 promoter-chloramphenicol acetyltransferase (CAT) reporter vector

The 5′-flanking region (−918 to +30 bp) was isolated from human genomic DNA by using an Advantage® Genomic PCR Kit (Cat. No. 639103 &639104, Clontech Laboratories, Inc, Mountain View, CA 94043). The product obtained was cleaned and sequenced as described previously [98,99]. A construct of −918 bp was prepared by ligating it to basic pCAT vector (Promega) using the *SacI* and *XhoI* sites. The plasmid was amplified and sequenced. Primers were as follows: Sense; 5′-GACAGAGTT*GAGCTC*CACACAG-3′; and antisense; 5′-CACGTC*CTCGAG*AAGCAGAC-3′ [10].

### Cotransfection and promoter activity assay

The CAT assay was performed using a CAT-ELISA kit (Roche Diagnostic Corporation, Indianapolis, IN, USA). Cells were transfected with reporter construct (pCAT-Prdx6), and treated with different concentrations of Sp1 inhibitor, Mithramycin A (Cat. No. M6891, Sigma Aldrich, St. Louis, MO, USA). After 72 h of incubation, cells were harvested, extracts were prepared and protein was normalized. CAT-ELISA was performed to monitor CAT activity in accordance with the manufacturer’s instructions. A_405_ was measured using a microliter plate ELISA reader. Transactivation activities were adjusted for transfection efficiencies using GFP values cotransfected during transfection assays.

### Induction of ultraviolet (UV) B induced stress

For UVB treatment, mLECs and SRA-hLECs were pre-cultured for 16h in 100mm petri dishes with DMEM-10% and 15% FBS. The medium was replaced with phosphate buffered saline (PBS, pH 7.2) and the plates containing the monolayers were exposed to UVB using UV-lamp emitting 270–320 nm peaking at 302 nm wavelength (UVP, Upland, CA, USA). The energy actually incident onto the working area was measured by a UVX Radiometer (UVP Inc., Upland, CA) and expressed in J/m^2^. The UV dosage of J/m^2^ was selected on the basis of results from our previous work [19]. After irradiation, PBS was withdrawn and fresh medium was added. At different time points protein was isolated and processed for Sandwich and Sumo1 specific ELISA assay to measure total Sp1 and Sumoylated Sp1 levels, respectively.

SRA-hLECs were cotransfected with pCl-neo-HA Sp1 WT or pCl-neo-HA Sp1 K16R along with either pEGFP-Vector or pEGFP-Sumo1. 48h later, the medium was replaced with phosphate buffered saline (PBS, pH 7.2) and the plates containing the monolayers were exposed to UVB as indicated. At intervals of 8 and 24 h later, ROS and MTS assays were performed to monitor the levels of ROS and cell viability, and the percentage of ROS and cell survival levels were then calculated for each group.

For another set of experiments, *Prdx6^-/-^* mLECs and SRA-hLECs were pre-cultured for 16h in 100mm petri dishes with DMEM-10% and 15% FBS. Cells were washed with PBS and transduced with TAT-HA-Prdx6 WT or its mutant TAT-HA-Prdx6 K122/142R for 3h; the medium was replaced with phosphate buffered saline (PBS, pH 7.2), and the plates containing the monolayers were exposed to UVB as indicated. After irradiation, PBS was withdrawn and fresh medium was added. Similar treatment was repeated for 2-3 days as indicated in the figures.

### Induction of hydrogen peroxide (H_2_O_2_) induced stress

For H_2_O_2_ treatment, mLECs and SRA-hLECs were pre-cultured for 16h in 100mm petri dishes with DMEM-10% and 15% FBS. Cells were washed twice with PBS, and the medium (0.2% BSA+ DMEM) was replaced with predefined concentrations of H_2_O_2_. At different time intervals protein was isolated and processed for Sandwich and Sumo1 specific ELISA assay to measure total Sp1 and Sumoylated Sp1 levels, respectively.

SRA-hLECs were cotransfected with pCl-neo-HA Sp1 WT or pCl-neo-HA Sp1 K16R along with either pEGFP-Vector or pEGFP-Sumo1. 48h later, the medium (0.2% BSA+ DMEM) was replaced with predefined concentrations of H_2_O_2_. At intervals of 8 and 24h later, ROS and MTS assays were performed to monitor the levels of ROS and cell viability, and the percentage of ROS and cell survival levels were calculated for each group.

For another set of experiments *Prdx6^-/-^* mLECs and SRA-hLECs were pre-cultured for 16h in 100mm petri dishes with DMEM-10% and 15% FBS. Cells were washed with PBS and transduced with TAT-HA-Prdx6 WT or its mutant TAT-HA-Prdx6 K122/142R for 3h, and the medium (0.2% BSA+ DMEM) was replaced with predefined concentrations of H_2_O_2_. Similar treatment was repeated for 2-3 days as indicated in figures.

### Extraction of nuclear and cytosolic fraction

Nuclear extract was prepared following the method of Sambrook et al. (18) with certain modifications. Briefly, LECs (1 X 10^6^) were cultured in 100-mm plates. The cells were washed gently with chilled phosphate-buffered saline (pH 7.4). Cells were collected by centrifugation using a micro-centrifuge and resuspended in 5 pellet volumes of cytoplasmic extract buffer [(10 mM HEPES (adjusted pH at 7.9), 10mM KCl, 0.1 mM EDTA, 0.4% (v/v) Nonidet P-40, 0.5mM phenylmethylsulfonyl fluoride (PMSF), 1mM DTT and Protease inhibitor]. After a short incubation on ice and centrifugation (4ºC) at 10000 rpm for 10min, the cytoplasmic extract was transfer in fresh tube from the pellet. Following careful washing with cytoplasmic extract without detergent (Nonidet P-40), the fragile nuclei were resuspended in nuclear extract buffer [(20 mM HEPES (adjusted pH at 7.9), 0.4M NaCl, 1mM EDTA, 10% (v/v) glycerol, 1mM DTT, 0.5mM PMSF and Protease Inhibitor] and incubated 2h at 4ºC continuous vortexing. Finally, the extract was spin at 14,000 rpm for 15 min to pellet the nuclei. After centrifugation, the nuclear extract was transferred and aliquoted in fresh tubes, and individual aliquots were stored at −70 °C to avoid repeated freezing and thawing of the preparation. Protein was estimated according to the Bradford protein assay and/or Pierce^TM^ BCA Protein assay methods and extract was used for experiment as required.

### Plasmids or constructs detail

**Construction of pEGFP-Sumo1:** For eukaryotic expression, the full length of Sumo1 cDNAwas subcloned into pEGFP-C1 vector. The coding region of Sumo1 was amplified by PCR from human lens cDNA library using forward (5’-CCGTCGACATGTCTGACCAGGAG-3’) and reverse primer (5’-TCGGATCCGTTTTGAACACCACA-3’) with restriction enzyme sites, Sa*lI* and Bam*HI*. The PCR product was digested and ligated into pEGFP vector.

pFlag-Senp1 was a generous gift from Dr. E. Yeh (University of Texas MD Anderson Cancer Center, Houston, TX, USA). All the Transfection experiments were carried out either with Superfactamine Reagent (Invitrogen, Carlsbad, CA, USA) or using the Neon Transfection system (Invitrogen). HA-tagged Sp1 and deleted construct [pClneo-HA-Sp1 (1-293)] was a gift from Dr. Hans Rotheneder (University of Vienna, Austria) [100]. pClneo-HA-Sp1K16R and pClneo-HA-Sp1 (1-293) K16R generated by mutagenesis.

### Site-directed mutagenesis (SDM)

PCR-based site-directed mutagenesis was carried out using the QuikChange^TM^ lightning site-directed mutagenesis kit (Agilent Technologies; Catalog No. 210518), following the company's protocol. Briefly, amino acid exchanges K16R were generated by point mutations in the pCl-neo-HA-Sp1 constructs. The following complementary primers, forward primer: 5′-GCTGTGGTGAGGATTGAAAAAGGAGTTGGTGGC-3′ and reverse primer: 5’-GCCACCAACTCCTTTTTAAATCCTCACCACAGC-3’ were used (changed nucleotides are in boldface type and underlined).

Epicurean Coli XL1-Blue super-competent cells (Stratagene) were transformed with resultant plasmid. The plasmid was amplified, and the mutation was confirmed by sequencing as described previously [101].

### *In vivo* Sumoylation assay

SRA-hLECs was transfected as indicated in figures. After 48h, nuclear extracts (as mentioned above) or total cell lysates were prepared in IP lysis/wash buffer (0.025 m Tris, 0.15 m NaCl, 0.001 m EDTA, 1% NP-40, 5% glycerol, pH 7.4 plus 5 μm MG132 and 30 μm *N*-ethylmaleimide was added, as provided in the Pierce Classic IP Kit (catalog number 26146; Pierce, Rockford, IL, USA), in accordance with the manufacturer's instructions. Nuclear extracts diluted in lysis buffer or total cell lysates were incubated with 4μg of anti-Sp1 (sc-17824, Santa Cruz)/anti-HA (ab18181, Abcam) monoclonal serum/800 μg of protein in IP lysis buffer provided in the IP kit (Pierce) and were rotated at 4°C overnight. That was followed by the addition of 20 μL of Protein A/G plus Agarose beads and further rotation for 4h at 4 °C. The immunoprecipitates were collected by centrifugation and washed several times with wash buffer and 1X conditioning buffer before being boiled in SDS sample buffer. Next, 10% input and IP samples were resolved on 4–20% SDS/PAGE and analyzed by western blotting using anti-Sp1, anti-Sumo1 or anti-HA rabbit polyclonal antibodies.

### Gel-shift and depletion assays

Gel-shift assay was carried out using nuclear extracts isolated from pEGFP-Vector or pEGFP-Sumo1 overexpressed SRA-hLECs to determine DNA binding of Sp1 to their respective elements present in the Prdx6 promoter. Oligonucleotides containing Sp1 binding sites elements were commercially synthesized (Invitrogen). Sequences were annealed and labeled with [ɤ-^32^P] ATP using T4 polynucleotide kinase (New England Biolabs, Inc.). The binding reaction was performed in 20µl buffer containing 20mM Tris-HCl (pH 8.0), 75mM KCL, 5% glycerol, 50µg/ml bovine serum albumin (BSA), 0.025% nonidet NP-40, 1mM EDTA, 5mM DTT, and 1µg of poly (dI/dC). The labeled probe [5fmol (1000cpm)] was incubated on ice for 30min with 5µg of nuclear extract. Samples were loaded on a 5% polyacrylamide gel in 0.5X TBE buffer and auto-radiographed.

### Cell survival assay (MTS assay)

A colorimetric MTS assay (Promega, Madison, WI, USA) was performed as described earlier [16,94,102]. This assay of cellular viability uses 3-(4, 5-dimethylthiazol-2-yl)-5-(3-carboxymethoxyphenyl)-2 to 4-sulphophenyl) 2H-tetrazolium salt; MTS and an electrone coupling reagent (Phenazine ethosulfate; PES).PES has enhanced chemical stability, which allows it to be combined with MTS to form stable solution. Assays are performed by adding MTS reagent directly to culture cells, incubating for 1-4h and then recording absorbance at 490nm with a 96-well plate reader, Spectra Max Gemini EM (Mol. Devices, Sunnyvale, CA). Results were normalized with absorbance of the untreated control(s).

### Cycloheximide (CHX), a translational blocker treatment

To inhibit translation/ protein synthesis, transfected cells as indicated were treated with 0–40 *μ*g/ml CHX for 0h-48h. CHX inhibitor (Catalog no. C4859) was purchased from Sigma-Aldrich. On the day of termination of experiment, total cell lysate prepared and immunoblotted with specific antibodies as indicated in figure and legends.

### Protein expression analysis

Cell lysates of LECs were prepared in ice-cold radioimmune precipitation buffer and protein blot analysis was performed as described previously [46,103,104]. The membranes were probed with anti-HA (ab 18181 and ab9110, Abcam^®^, Cambridge, MA, USA), Anti-Sp1( Anti-Prdx6 antibody (LF-PA0011 and LF-MA0018, Ab Frontier, South Korea), or β-actin (A2066, Sigma-Aldrich, St. Loius, MO, USA)/Tubulin (ab7291, Abcam^®^, Cambridge, MA, USA) as internal control to monitor those protein expressions. After secondary antibody (sc-2354 and sc-2768, Santa Cruz Biotechnology, Dallas, TX, USA), protein bands were visualized by incubating the membrane with luminol reagent (sc-2048; Santa Cruz Biotechnology, Dallas, TX, USA) and images were recorded with a FUJIFILM-LAS-4000 luminescent image analyzer (FUJIFILM Medical Systems Inc., Hanover Park, IL, USA).

### TAT-HA-Prdx6 recombinant protein purification

A full-length cDNA of Prdx6 was isolated from a human LEC cDNA library using Prdx6-specific sense (5′-GTCGCCATGGCCGGAGGTCTGCTTC-3′ containing *Nco*I site) and antisense primer (5′-AATTGGCAGCTGACATCCTCTGGCTC-3′). The PCR products were purified by preparative agarose gel electrophoresis. The purified products were ligated into a TA-cloning vector (Invitrogen) and then transformed into a competent cell and the plasmids of selected colonies were purified. The purified TA vector containing Prdx6 cDNA was digested with *Nco*I and *Eco*RI and then subcloned into a pTAT-HA expression vector (a kind gift of Dr S. F. Dowdy, Howard Hughes Medical Institute, University of California, San Diego, CA) that had been digested with the same restriction enzymes. Wild type (WT) TAT-HA- Prdx6 was then mutated at K (lysine) 122/142 (arginine) R by using SDM kit. Recombinant protein was purified using QIAexpress^®^ Ni-NTA Fast Start kit column (Qiagen Inc., Valencia, CA, USA). The host *Escherichia coli* BL21 (DE3) was transformed with pTAT-HA-Prdx6 and the transformants were selected on a LB plate with ampicillin. The selected colonies were cultured in 10 mL of LB medium containing ampicillin at 37 °C with shaking at 200 r.p.m. overnight. After incubation, 10 mL of the overnight cultures were combined with 250 mL of pre-warmed media (with ampicillin) and then grown at 37 °C with vigorous shaking until *D*_600_ of 0.6–0.8 was reached, and then isopropyl thio-β-d-galactoside was added to a concentration of 1 mm and the incubation was continued for 4–5 h. Cells were harvested by centrifugation at 4000 *g* for 20 min. Pellets were suspended in 10 mL of lysis buffer (50 mm NaH_2_PO_4_, 50 mm NaCl and 10 mm imidazole, pH 8.0) containing lysozyme and Benzonase^®^ Nuclease (Qiagen Inc., Valencia, CA, USA) and incubated for 30 min on ice. The suspension was then centrifuged at 14 000 *g* for 30 min. Supernatant was added to the Ni-NTA fast start column and allowed to drain before washing twice with 4 mL of wash buffer (50 mm NaH_2_PO_4_, 50 mm NaCl and 20 mm imidazole, pH 8.0), followed by elution with 1 mL of elution buffer (50 mm NaH_2_PO_4_, 50 mm NaCl and 250 mm imidazole, pH 8.0).

### Statistical methods

For all quantitative data collected, statistical analysis was conducted by Student’s *t test* and /or one-way ANOVA when appropriate, and was presented as mean ± S.D. of the indicated number of experiments. A significant difference between control and treatment group was defined as *P* value of < 0.05 and 0.001 for two or more independent experiments.

## Supplementary Material

Supplementary Figures
