# Supplementary material for: Sumoylation-deficient Prdx6 repairs aberrant Sumoylation-mediated Sp1 dysregulation-dependent Prdx6 repression and cell injury in aging and oxidative stress
Source: Aging (Albany NY). 2018 Sep 12;10(9):2284–315. doi: 10.18632/aging.101547 (PMC6188488; doi:10.18632/aging.101547)
Supplement: Supplementary Figures [file aging-10-101547-s001.pdf]

SUPPLEMENTARY FIGURES

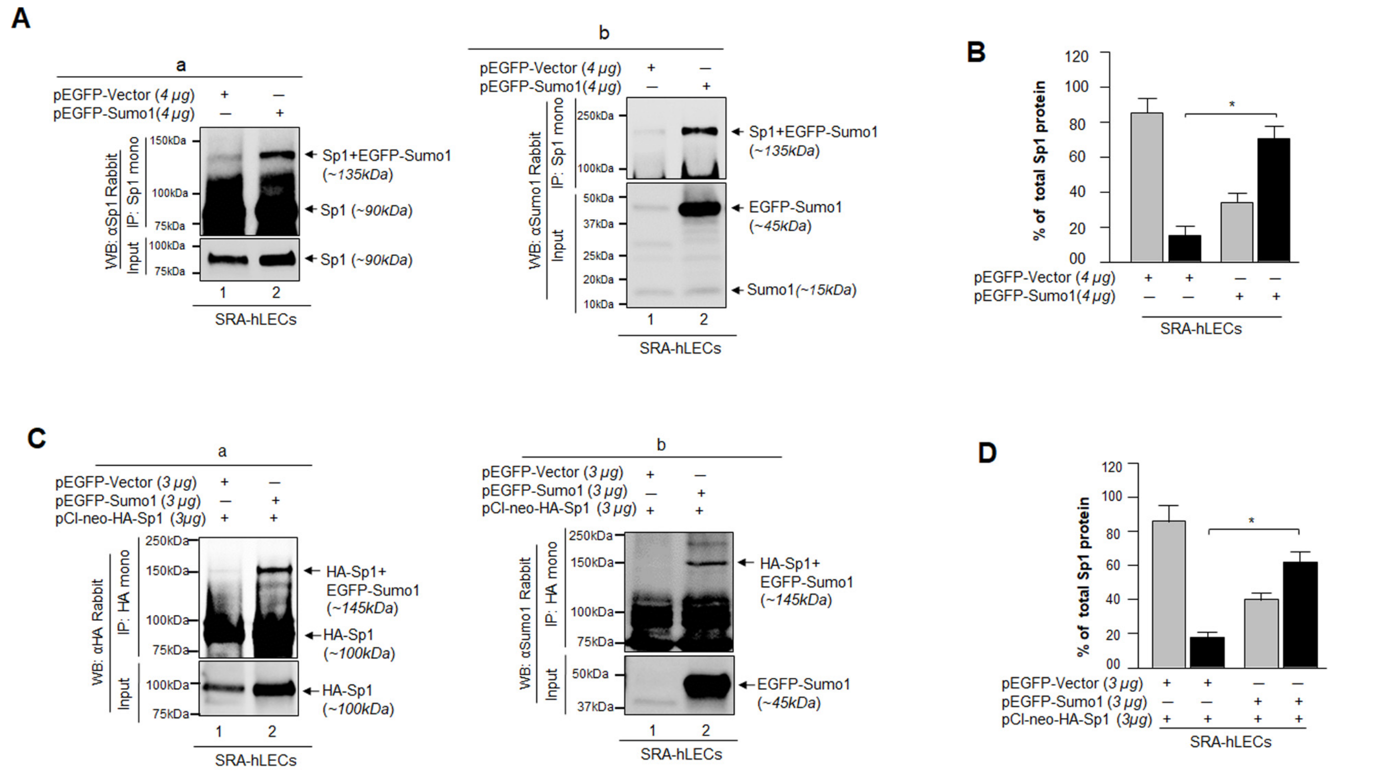

Supplementary Figure 1. Intrinsic or extrinsic Sp1 was Sumoylated in LECs *in vivo*. (**A**) Intrinsic Sp1 protein is a substrate for Sumo1 *in vivo*. SRA-hLECs (1X10^{6}) were overexpressed with pEGFP-Vector or pEGFP-Sumo1. Cells transfected with pEGFP-Vector served as control (**Aa** and **Ab**, Lane 1). 48h after transfection, nuclear extracts were prepared and subjected to immunoprecipitation (IP) using anti-Sp1 monoclonal antibody. Input and IP samples were resolved on 4–20% SDS-PAGE and immunoblotted with anti-Sp1 (**Aa**) or anti-Sumo1 (**Ab**) rabbit polyclonal antibodies and visualized as described in Materials and Methods. IP experiments revealed the presence of two bands with Sp1 antibody: ~90kDa (unSumoylated endogenous Sp1), and ~135kDa (endogenous Sp1 Sumoylated by pEGFP-Sumo1), indicating that Sp1 may contain a single site for Sumo1 protein. (**B**) Sensitive Sp1 Sandwich/Sumo1-ELISA assays validated that intrinsic Sp1 was Sumoylated, and showed that a fraction of endogenous Sp1 was present in Sumoylated form. SRA-hLECs were transfected with pEGFP-vector or pEGFP-Sumo1. 48h later nuclear extracts were prepared and submitted to Sp1 sandwich/Sumo1-ELISA assays to check the total Sp1 (IP: Sp1) protein and Sumoylated Sp1 (IP: Sp1) protein. Sumoylated Sp1 protein was subtracted from total Sp1 protein, presenting as deSumoylated Sp1 (gray bars) and Sumoylated Sp1 (black bars) forms. The data represent mean ± SD from three independent experiments (*p<0.001). (**C**) SRA-hLECs (1.2X10^{6}) were cotransfected with pCl-neo-HA-Sp1 (3μg) along with pEGFP-Sumo1 (3μg) or pEGFP-vector (3μg). After 48h, total cell lysates were prepared and subjected to immunoprecipitation (IP) using anti-HA monoclonal or control IgG antibodies. 10% Input and IP samples were resolved onto 4-20% SDS-PAGE and immunoblotted with anti-HA (**Ca**) and anti-Sumo1 (**Cb**) rabbit polyclonal antibodies. Sumoylated band was visualized with both antibodies at ~145kDa (pEGFP-Sumo1 [~45kDa] plus pHA-Sp1 [~100kDa]; lane 2). In input, HA-Sp1 was seen with anti-HA antibody and EGFP-Sumo1 with anti-Sumo1 antibody. (**D**) Sensitive Sp1 sandwich/Sumo1-ELISA assays validated that extrinsic Sp1 was Sumoylated. SRA-hLECs were transfected with pEGFP-vector plus pHA-Sp1 or pEGFP-Sumo1 plus pHA-Sp1. Total cell lysates were prepared and used to perform Sp1 sandwich/Sumo1-ELISA. Sumoylated Sp1 protein (IP: HA) was subtracted from total Sp1 (IP: HA) protein, presenting as deSumoylated Sp1 (gray bars) and Sumoylated (black bars) forms. The data represent the mean ± SD of three independent experiments; *p<0.001.

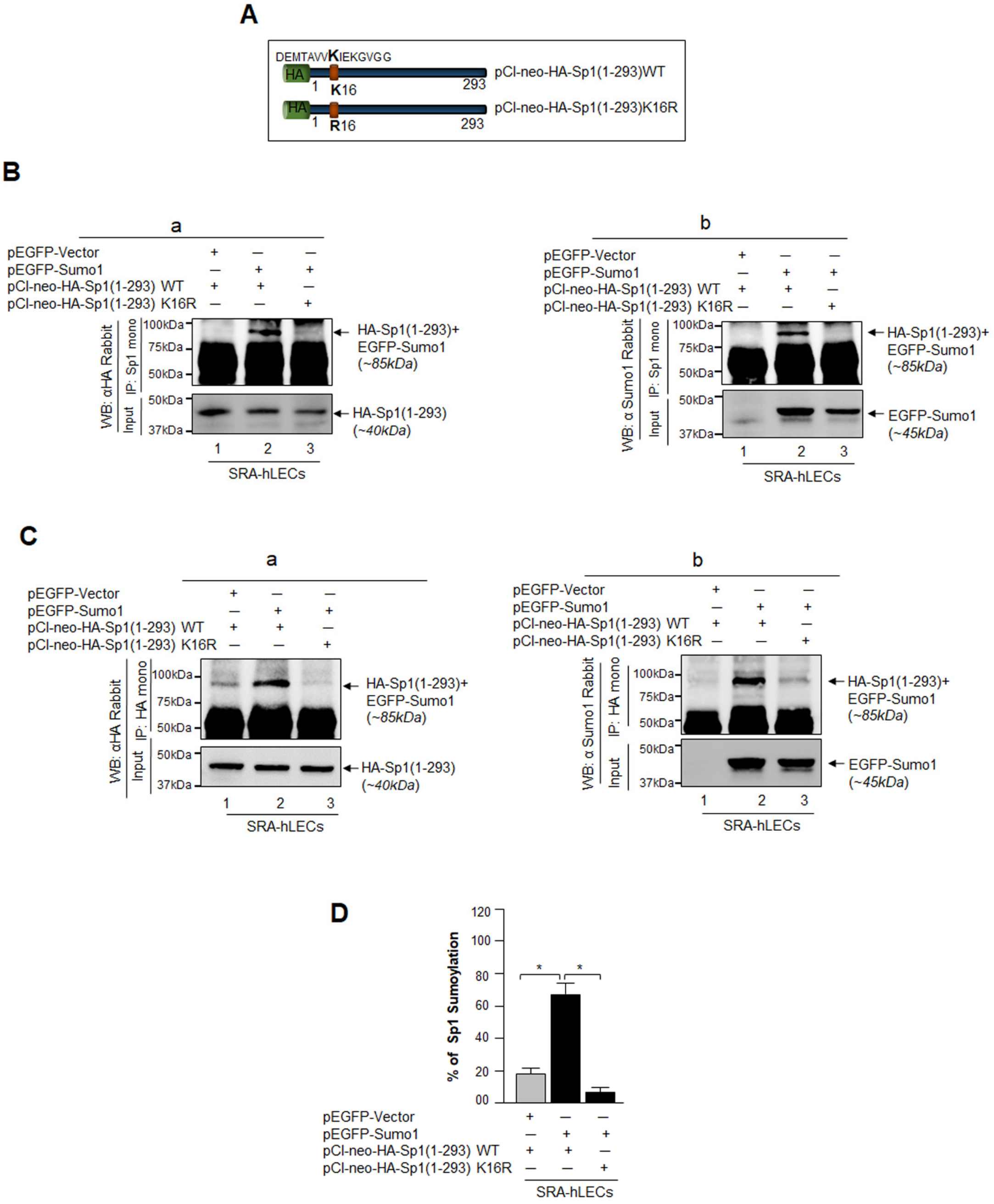

Supplementary Figure 2. Sumo1 was conjugated to lysine K16 of Sp1 *in vivo*. (**A**) Top panel, a diagrammatic illustration of Sp1 deletion construct with 293aa, pCl-neo-HA-Sp1(1-293) WT , Sp1 Full construct pCl-neo-HA-Sp1 and their mutant (at K16 to R16) plasmids. (**B and C)** SRA-hLECs (1.2X10^{6}) were cotransfected with pCl-neo-HA-Sp1 (1-293) WT (3µg) plus pEGFP-Vector (3µg) or pCl-neo-HA-Sp1 (1-293) WT (3µg) plus pEGFP-Sumo1 (3µg) or pCl-neo-HA-Sp1 (1-293) K16R (3µg) plus pEGFP-Sumo1 (3µg). 48h later, cellular extracts were prepared and subjected to IP using anti-Sp1 (B) or anti-HA (C) monoclonal antibodies and immunoblotted as indicated. Single exogenous Sumoylated band was observed at ~85kDa [~40kDa, pCl-neo-HA-Sp1 (1-293) WT + pEGFP-Sumo1 (~45kDa)] in pCl-neo-HA-Sp1 (1-293) WT plus pEGFP-Sumo1 transfected (**B** and **C**, lane 2) cells with anti-HA and anti-Sumo1 rabbit polyclonal antibodies. No Sumoylated bands were detected in pCl-neo-HA-Sp1(1-293)WT plus pEGFP-Vector (B and C, Lane 1) and pCl-neo-HA-Sp1(1-293) K16R (B and C, Lane 3) transfected cells. **(D)** An *in vivo* Sumoylation ELISA assay was done according to the manufacturer's protocol (EpiQuik^{TM}). SRA-hLECs were transfected with pCl-neo-HA-Sp1(1-293)WT plus pEGFP-Vector or pCl-neo-HA-Sp1(1-293)WT plus pEGFP-Sumo1 or pCl-neo-HA-Sp1(1-293)K16R plus pEGFP-Sumo1. 48h later, total cell lysates were prepared and processed for Sumo1-ELISA assay to measure Sumoylated form of Sp1. Data represent mean ± SD from three independent experiments. pCl-neo-HA-Sp1 (1-293)WT plus pEGFP-Vector vs pCl-neo-HA-Sp1(1-293) WT plus pEGFP-Sumo1 vs pCl-neo-HA-Sp1(1-293) K16R plus pEGFP-Sumo1 (*p<0.001).
